# Pleiotropic physiological functions of Piezo1 in human body and its effect on malignant behavior of tumors

**DOI:** 10.3389/fphys.2024.1377329

**Published:** 2024-04-16

**Authors:** Yihan Zhang, Wen Zou, Wenlei Dou, Hongliang Luo, Xi Ouyang

**Affiliations:** ^1^ Department of Gastrointestinal Surgery, The Second Affiliated Hospital of Nanchang University, Jiangxi Medical College, Nanchang University, Nanchang, China; ^2^ The Second Clinical Medicine School, Jiangxi Medical College, Nanchang University, Nanchang, China

**Keywords:** Piezo1, tumor, initiation and progression, metastasis, therapeutic target

## Abstract

Mechanosensitive ion channel protein 1 (Piezo1) is a large homotrimeric membrane protein. Piezo1 has various effects and plays an important and irreplaceable role in the maintenance of human life activities and homeostasis of the internal environment. In addition, recent studies have shown that Piezo1 plays a vital role in tumorigenesis, progression, malignancy and clinical prognosis. Piezo1 is involved in regulating the malignant behaviors of a variety of tumors, including cellular metabolic reprogramming, unlimited proliferation, inhibition of apoptosis, maintenance of stemness, angiogenesis, invasion and metastasis. Moreover, Piezo1 regulates tumor progression by affecting the recruitment, activation, and differentiation of multiple immune cells. Therefore, Piezo1 has excellent potential as an anti-tumor target. The article reviews the diverse physiological functions of Piezo1 in the human body and its major cellular pathways during disease development, and describes in detail the specific mechanisms by which Piezo1 affects the malignant behavior of tumors and its recent progress as a new target for tumor therapy, providing new perspectives for exploring more potential effects on physiological functions and its application in tumor therapy.

## 1 Introduction

Nowadays, the main ways of tumor treatment include radiotherapy, chemotherapy, surgery, and targeted therapy (Chen et al., 2023). Of these methods, radiotherapy and chemotherapy are the most common non-surgical treatments, and they are often used in combination with surgery. Radiotherapy uses high-energy radiation to kill cancer cells, which can accurately target the tumor area and cause minimal damage to surrounding normal tissues, making it suitable for the treatment of many tumors. Chemotherapy uses drugs to interfere with the growth of tumor cells, and chemotherapy agents are usually administered orally or intravenously. Chemotherapy is also suitable for the treatment of a variety of malignant tumors and some benign tumors. Surgery is one of the important ways to treat malignant tumors. Open surgery or minimally invasive surgery can be selected according to the specific situation of the patient, and resection of the tumor can achieve the treatment goal. However, the toxicity of traditional therapy seriously affects the patient’s tolerance and compliance (Mun et al., 2018). Drug resistance of tumor cells is one of the main reasons for the failure of traditional therapies and targeted therapies can help break through this situation (Holohan et al., 2013).

Targeted therapy is a kind of treatment that has emerged in recent years. By targeting specific tumor targets, targeted therapy can effectively reduce the side effects of treatment and improve the therapeutic effect (Singh, 2023). With the rapid development of precision medicine, molecular targeted therapy has been widely used in clinical tumor treatment because of its few side effects and high accuracy compared with traditional strategies (Zhang XN. et al., 2023).

The development of the tumor is highly correlated with the physiological state of the tumor microenvironment (TME). Although tumors from the same or different anatomical locations exhibit heterogeneity, common features can be found in mature TME of epithelial metaplastic tumors. TME predominates in terms of prognosis and influences the efficacy of anti-tumor therapies. Targeting TME more efficiently is of great significance for treating tumors (Roma-Rodrigues et al., 2019). This kind of targeted therapy has already achieved a lot in tumors. For example, targeted therapy has become an indispensable part of breast cancer treatment, and HER2 has been widely applied in the clinical treatment of breast cancer and achieved remarkable results (Zhang XN. et al., 2023). Similarly, in the treatment of non-small cell lung cancer, combination targeted therapy has more advantages than traditional chemotherapy and anti-PD-1 immunotherapy alone (Lv et al., 2019). For infants and young children, traditional chemotherapy and radiation therapy may have toxic side effects on brain development, while targeted therapy with biological agents has been proven to reduce these toxic side effects and have the same or higher efficacy (Nageswara Rao et al., 2012). Recently, it has been found that transforming growth factor-β (TGF-β) affects the effects of tumor immunotherapy, and targeted treatment with TGF-β can effectively improve the survival time of patients and reduce side effects (Batlle and Massagué, 2019). The achievements of targeted therapies are numerous.

The momentous discovery in 2010 of the function of mechanosensitive ion channels protein 1 (Piezo1) and protein 2 (Piezo2) channels not only won the 2021 Nobel Prize in Physiology or Medicine but also brought the understanding of mechanical transduction a step closer. Mechanical stimulation drives many physiological processes, including the perception of pain, temperature, pressure and the regulation of vasoconstriction and relaxation. Studies have confirmed that Piezo1 is necessary for mechanically activating cation channels (Coste et al., 2010). Piezo1 channels are proteins associated with mechanical stress-sensitive ion channels. It is widely present in many cell types and plays a key role in the regulation of the intracellular and extracellular environment. Piezo1 has special ion channel characteristics, that is, once the cell senses mechanical stimulation, it immediately opens the ion channel and non-selectively allows cations, especially Ca^2+^, to pass through (Qin et al., 2021). This influx and outflow of cations in turn affects downstream signal transduction, thereby promoting internal and external cell signal transduction (Yarishkin et al., 2021). It is precisely because of this characteristic that Piezo1 can serve as a bridge connecting mechanical forces and biological signals, and act as a bio-mechanical converter to mediate these mechanical reactions, affecting various cellular physiological activities (Köster et al., 2022), which are closely related to various pathological conditions such as idiopathic polycythemia (Filser et al., 2021) and renal fibrosis (Zhao X. et al., 2022). In addition, the activity of Piezo1 channels is regulated by mechanical stress rather than chemical signals, and this unique characteristic has attracted widespread attention in the fields of mechanical sensing, cell migration, hemorheology, and pain perception (Kim et al., 2012; Beech and Kalli, 2019a; Syeda, 2021; Mukhopadhyay et al., 2024). In recent years, several studies have linked mutations in Piezo1 to the onset and development of a variety of diseases (Dienes et al., 2023). For example, the Piezo1 gene plays an important regulatory role in dental pulp stem cells, suggesting that it may be involved in the development of pulpitis and other oral diseases (Xing et al., 2022). In addition, studies have found that the Piezo1 gene plays an important role in T cell receptor signal transduction, revealing its possible association with autoimmune diseases (Lukacs et al., 2015). Similarly, the activation of Piezo1 can cause relaxation of the pubic arteries and corpus cavernosum (Dela Justina et al., 2023), as well as regulate the migration of microglia and immune responses (Zhu et al., 2023). It follows that Piezo1 is closely associated with cellular life and that the proper opening of the Piezo1 is important for the human body.

In recent years, an increasing number of studies have linked the Piezo1 channel with tumor development. Tumors are a serious threat to human health, and their development process involves the abnormal regulation of multiple cell signaling pathways. The research suggests that the Piezo1 channel plays an important regulatory role in tumor development. First, Piezo1 is linked to the ability of tumor cells to migrate and invade. Multiple studies have found that the Piezo1 channel can increase the migration and invasion ability of tumor cells, and correspondingly, inhibiting its activity can weaken this migration and invasion ability. For example, in gastric cancer, Piezo1 is a novel trilobal-family-1-binding protein that promotes cancer cell migration *in vitro* (Yang et al., 2014). In osteosarcoma, Piezo1-shRNA was found to inhibit invasion of osteosarcoma cells, and the Piezo1 protein may be a new, potential therapeutic target (Jiang et al., 2017). Second, overexpression of the Piezo1 channel was associated with the proliferation and growth of tumor cells. Some studies have found that suppressing the Piezo1 channel inhibits the proliferation and growth of tumor cells. In synovial sarcoma, Piezo1 is highly expressed in SW982 cells, and knocking it down affects cell viability, making it a potential target against synovial sarcoma (Suzuki et al., 2018). In gliomas, high Piezo1 expression is associated with reduced survival time and serves as a strong biomarker for poor prognosis in gliomas (Zhou et al., 2020). Finally, the Piezo1 channel has also been linked to the transformation of tumor cells and drug resistance. Knowing the important role of the Piezo1 channel in tumor development, the researchers began exploring using the Piezo1 channel as a new target for tumor treatment.

Some studies have found that inhibiting the activity or expression of the Piezo1 channel can inhibit the growth and metastasis of tumor cells and enhance their sensitivity to chemotherapy drugs or ultrasonic therapy. As a result, the Piezo1 channel could be a potential target for tumor therapy. A large number of other tumor developments are underway or have been found to be connected to Piezo1, and Piezo1 has great potential as an anti-tumor target.

## 2 Basic information on Piezo1

The family of Piezo channels, divided into Piezo1 and Piezo2, plays an irreplaceable part in the mechanical stimulation transduction in mammals. Research has found that these channels were evolutionarily conserved and played key roles in various mammalian physiology, including pain and temperature allometry, proprioception, regulation of blood pressure, and vascular development (Gottlieb et al., 2012; Xu, 2016). Although both Piezo1 and Piezo2 can convert physical mechanical stimuli into biochemical information between cells, Piezo2 is mainly expressed in cells of neuronal origin (Stewart and Davis, 2019). This paper focuses on the structure, characteristics, function, and distribution of Piezo1.

Piezo1, a mechanosensitive ion channel protein, is a large homotrimeric membrane protein capable of converting force into chemoelectric signals (Mulhall et al., 2023). Piezo1 was also found to undergo potential reversal at around 0 mV, and more specifically, Piezo1 undergoes voltage-dependent inactivation. Heterologous expression of this protein can also produce mechanical sensitivity in otherwise insensitive cells (Fang et al., 2021). Studies have shown that Piezo1 channels are able to open in response to mechanical force stimulation, resulting in an increase in ion flux. This mechanical sensitivity allows Piezo1 channels to sense and respond to extracellular physical forces, such as mechanical stretch and shear (Saotome et al., 2018). Piezo1 is distributed in plenty of tissues and cells, especially the muscle, heart, nervous system, and vascular system; Related animal experiments also detected increased Piezo1 expression in the epithelial tissues of the bladder, colon, kidneys, lungs and skin of mice. This side verifies the universality of Piezo1 distribution (Coste et al., 2010).

As for the structure of the Piezo1 protein, the Piezo1 that has now been discovered is quite complex and ingenious, with multiple domains. These different domains have a variety of functions, working together to maintain the body’s physiological processes, such as sensing mechanical forces and maintaining cell proliferation. Piezo1 consists of an N-terminal region comprising approximately 3,000 amino acids, a transmembrane region, and a short C-terminal region, where the transmembrane region contains multiple transmembrane domains. Each protomer contacts the central pore and cap domain near the C terminus, and projects a leaf of 36 transmembrane domains that extend outward and upward toward the N terminus (Mulhall et al., 2023). Among them, the central pore module includes the C-terminal extracellular domain, transmembrane inner helices and outer helices, and the intracellular C-terminal domain. The combination of the three C-terminal extracellular domains forms the extracellular cap domain (Fang et al., 2021). The transmembrane region contains multiple transmembrane domains that collectively form a tube through which ions can pass, generating an electric current. This transmembrane region can be further subdivided into two parts: the jumping helical structure and the membrane insertion region. The jumping helices represent a unique topology with 38 transmembrane helices, forming an autotrimer propeller-shaped structure. This structure consists of a central ion-conducting hole and three peripheral mechanical sensing blades. Notably, the abnormally curved transmembrane regions of these three peripheral mechanical sensing blades create a nanobowl configuration (Jiang Y. et al., 2021). Recent research has revealed that both lipids and modified lipid bilayer compounds play a regulatory role in Piezo1, while the sufficient rigidity of the propeller like structure can bend the surrounding membrane to form a dome (Jiang Y. et al., 2021), which subsequently affects the components of the extracellular matrix (ECM) and the cytoskeleton (Vasileva and Chubinskiy-Nadezhdin, 2023).

In addition, Piezo1 protein encompasses several extracellular domains integral to its function. The switching of Piezo1 ion channels has been found to be influenced by external mechanical stimuli. For instance, the mechanosensitive protein Piezo1 plays a crucial role in regulating bone homeostasis through osteoblast-osteoclast crosstalk (Cai et al., 2023). When Piezo1 is activated, it is these extracellular domains that are initially affected by mechanical force. This force is then transmitted to the transmembrane domain, leading to the opening of channels through which ions can pass (Guo and MacKinnon, 2017). This mechanism elucidates the relationship between the opening of Piezo1 channels (Guo and MacKinnon, 2017; Saotome et al., 2018). In summary, the Piezo1 channel employs a mechanical transduction and ion conduction module to carry out its functions of sensing mechanical forces, gating, and facilitating ion conduction (Zhao F. et al., 2022).

**FIGURE 1:**
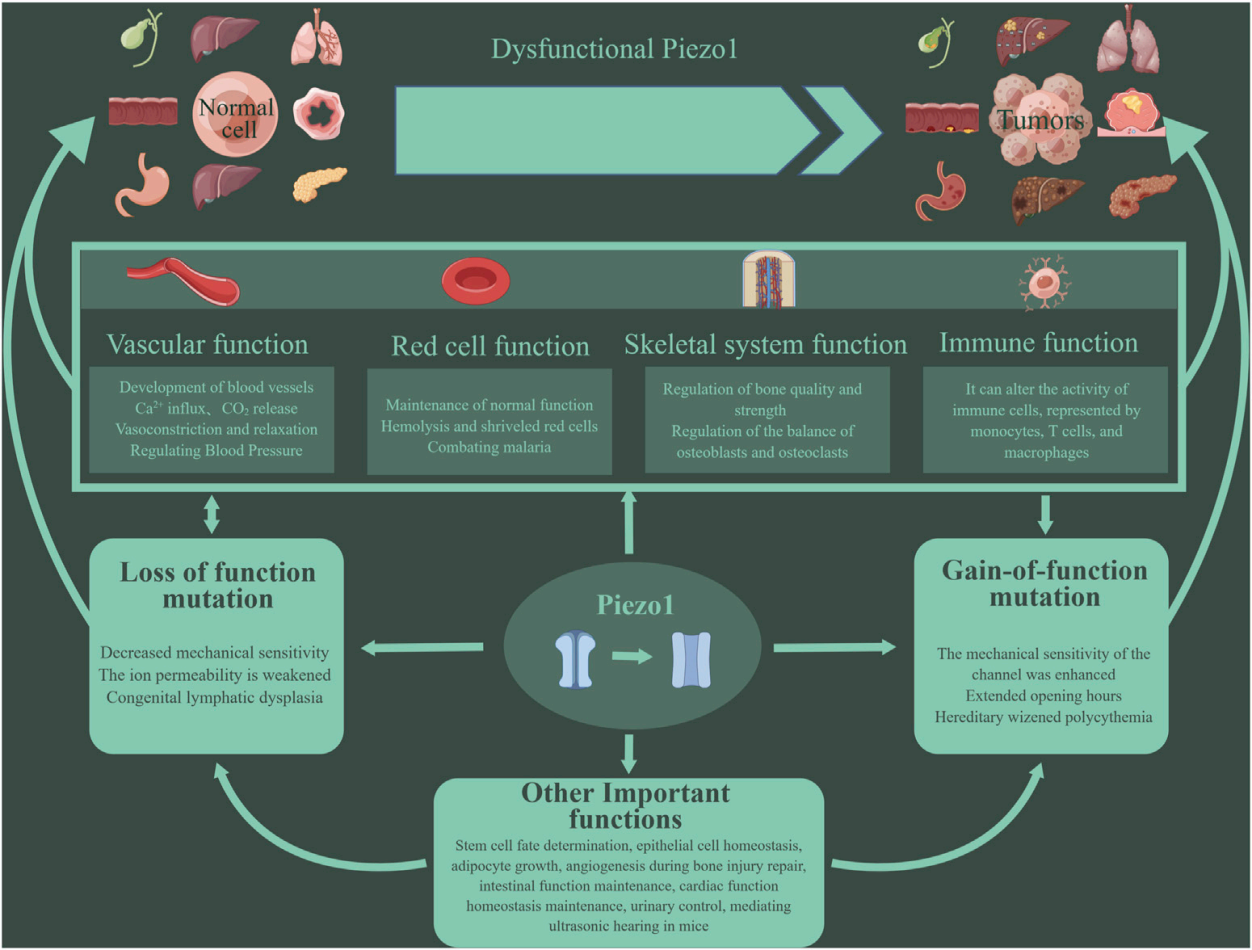
Piezo1 has multiple functions. Piezo1 can affect the cardiovascular system, red blood cell function, bone repair and reconstruction, immune function, etc. More and more studies have shown that the abnormal function of Piezo1 is related to the occurrence and development of tumors. Mutations in the Piezo1 gene can lead to inhibition or enhancement of Piezo1 function, eventually leading to various pathological changes. Such mutations are divided into two types: loss- and gain-of functions.

The novel and intricate structure of the Piezo1 protein holds the potential to inspire new avenues for drug development and the treatment of diseases. Due to its unique configuration, Piezo1 possesses the capability to detect and respond to various mechanical stimuli, including extracellular pressure, shear force and hydrostatic pressure. This mechanical sensing ability regulates the opening of ion channels, facilitating the flow of ions into and out of cells. Consequently, it initiates physiological cellular responses, such as muscle contraction, vasodilation, and nerve signaling. The alterations in physiological functions resulting from Piezo1 activation can be illustrated as follows (Figure 1).

### 2.1 Cardiovascular system

Piezo1 can be assembled into a transmembrane triple helix that combines fine force sensing with regulated calcium influx. Accumulating evidence suggests that they have important roles in vascular permeability and remodeling, blood pressure regulation, insulin sensitivity, endothelial shear stress sensing and secretion, nitric oxide production, vascular tone, angiogenesis, and atherosclerosis (Beech and Kalli, 2019b).

**FIGURE 2:**
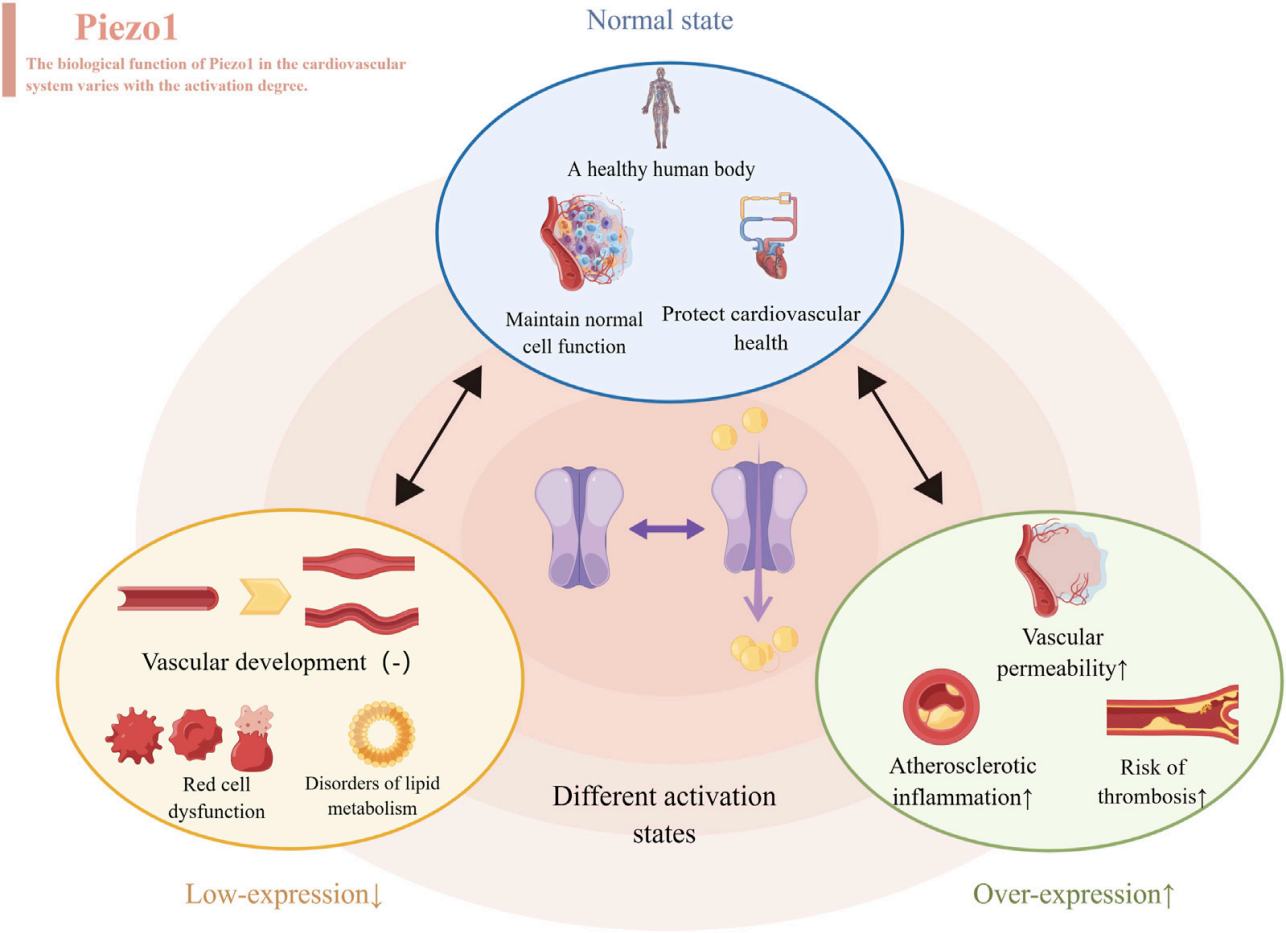
Different levels of Piezo1 expressed produce different effects, represented by the cardiovascular system. When normally activated, Piezo1 maintains normal cell function and protects cardiovascular health. When inhibitors of Piezo1 expression are used, they affect the development of blood vessels, disrupt the function of red blood cells and throw lipid metabolism out of kilter. When Piezo1 was overexpressed with an activator, it led to atherosclerosis, thrombosis and increased permeability of blood vessels.

First, Piezo1 channels are closely related to hemorheology. In the context of the cardiovascular system, experiments have demonstrated that Piezo1 and Piezo2 are essential for blood pressure regulation as baroreceptors (Ranade et al., 2014). It has been shown that in vascular endothelial cells, mechanical stimuli can activate Piezo1 channels, leading to regulation of blood vessel dilation and contraction (Beech and Kalli, 2019a). With the increase of fluid flow, Piezo1, a non-inactivated non-selective cation channel, can depolarize membrane potential to activate voltage-gated Ca^2+^ channels in adjacent vascular smooth muscle cells, causing vasoconstriction. Notably, it can raise blood pressure during physical activity by causing vasoconstriction, but this effect is not required during inactivity (Rode et al., 2017). Relevant animal experiments, such as zebrafish experiments, have shown that Piezo1 can be expressed in cardiomyocytes and has a homeostatic effect on cardiac function. The abnormal activity of Piezo1 channels has been implicated in the development and progression of several cardiovascular diseases, such as hypertensive thrombosis (Albarrán-Juárez et al., 2018a; Zhao et al., 2021). The opening of Piezo1 under high hydrostatic pressure has been found to disrupt pulmonary endothelial barrier function, remodel arteries during hypertension, and cause atherosclerosis during turbulence (Douguet et al., 2019). Genetic engineering experiments have shown that overexpression of Piezo1 in cardiomyocytes leads to severe cardiac hypertrophy and arrhythmias (Jiang F. et al., 2021). Similarly, artificial inhibition or activation of Piezo1 expression in mouse heart can lead to the interruption of Ca^2+^ and ROS signal transduction and promote the development of cardiomyopathy. In addition, Piezo1 expression or activity in the heart was increased under continuous stress and heart failure. Among them, the use of Piezo1 antagonists can inhibit excessive Piezo1 activity, providing therapeutic benefits (Rolland et al., 2023) (Figure 2).

### 2.2 Skeletal system

In the context of the skeletal system, Piezo1 plays a pivotal role in regulating the functions of bones, joints, tendons, and muscles, contributing to posture maintenance and power generation (Dienes et al., 2023). Numerous studies have highlighted the potential of the Piezo1 channel as a significant breakthrough in the treatment of diseases associated with muscular dystrophy and cartilage degeneration (Bagriantsev et al., 2014; Zhu et al., 2021). For instance, E756del is a genetic gain-function variant of Piezo1, and some research results indicate that the E756del variant of Piezo1 is acquired in mice and appears more frequently in sprinters compared to non-athletes. Additionally, animal experiments have demonstrated that mice with the R2482H mutation in Piezo1 exhibit improved jumping and elastic muscle functions (Nakamichi et al., 2022). Hence, it can be inferred that Piezo1 enhances skeletal muscle stretching function by improving tendon compliance. Furthermore, the application of a double-layer membrane loaded with Yoda1 not only prevents fibroblast entry but also, through Piezo1 activation, promotes bone formation and angiogenesis (Yang et al., 2023). Downregulating Piezo1 inhibits macrophage polarization towards the repair type, thereby affecting bone remodeling. Animal experiments have also shown that the downregulation of Piezo1 significantly reduces exercise-induced bone mass in mice (Cai et al., 2023). Notably, cartilage cells can discern and respond to varying levels of mechanical stimulation, potentially due to differences in mechanically sensitive ion channels, such as TRPV4, Piezo1, and Piezo2. TRPV4, Piezo1 and Piezo2 are capable of sensing different levels of repeated mechanical tensile stimuli, and experiments have indicated that tensile strength can influence Piezo’s expression. When cyclic tensile strains were increased to high strain levels of 13% and 18%, Piezo1 expression exhibited a significant increase, whereas this effect was not observed at low strain levels compared to unstretched controls. Unlike Piezo1, the primary function of Piezo2 is to sense harmful levels of repeated cyclic tensile strains loads (Du et al., 2020). It has been suggested that a synergistic interaction between Piezo1 and Piezo2 is essential for maintaining the functionality of joint cartilage (Zhong et al., 2018).

### 2.3 Red blood cells

In terms of the function of red blood cells, the activation of Piezo1 can promote the influx of extracellular Ca^2+^ into cells. Some studies have found that the activation of Piezo1 during platelet driven blood clot contraction may be caused by the compression and deformation of red blood cells. Antagonizing Piezo1 causes a decrease in clot contraction relative to the control group. Therefore, it can be inferred that the activation of Piezo1 by compressed and deformed red blood cells amplifies platelet contractility, forming a positive feedback mechanism during the contraction of blood clots (Evtugina et al., 2023). Piezo1 can not only affect calcium homeostasis but also directly intervene in red blood cell degradation by regulating iron metabolism. Activating Piezo1 can increase iron content in serum and the liver (Hanchard and Wonkam, 2021). Piezo1 was found to be strongly linked to a variety of anemia. For example, studies have found that HBS aggregation can activate Piezo1, thereby increasing intracellular calcium ion concentration, ultimately increasing the number of sickle red blood cells and promoting the progression of sickle cell anemia (Nader et al., 2023). Targeting Piezo1 may be a promising treatment for sickle cell anemia (Gibson and Stewart, 2023). In addition, mutations in the Piezo1 gene are closely related to the occurrence and development of type B marine anemia by affecting red blood cell function (Pinto et al., 2023). Because Piezo1 is the carrier of Er antigen, Piezo1 mutation is closely related to Er red blood cell antigen, which may explain Piezo1’s connection to neonatal hemolytic anemia (Karamatic et al., 2023). Interestingly, Piezo1 is related not only to anemia but also to the prevention of malaria. Polymorphisms and mutations in Piezo1 have been linked to malaria prevention. The reason is that Piezo1 prevents plasmodium falciparum from entering red blood cells, and E756del has been shown to prevent even severe malaria. Meanwhile, activating Piezo1 has been shown *in vivo* and *in vitro* to protect humans from malaria (Lohia et al., 2023).

### 2.4 Immunity

An increasing number of studies have found that mechanistically gated ion channels, especially Piezo1, play a key role in regulating the immune system (Hamza et al., 2021). In the case of immunity, bacteria cross the cell membrane as a mechanical signal, stretching the membrane to indirectly activate the channel Piezo1, which triggers changes in the body’s immune system (Giron-Ceron and Jaumouillé, 2023). The mechanical stimulation mediated by membrane folding upon entry activates Piezo1, which in turn increases the production of ATP. In addition, mechanically stimulating the physical signal to activate Piezo1 could lead to increased gene expression in immune and barrier pathways, ultimately leading to an immune response (Tadala et al., 2022). Among them, Piezo1 can alter the activity of immune cells such as macrophages, B cells and microglia. For macrophages, the mechanism may be that Piezo1 mediated mechanical sensitive signals can enhance the aerobic glycolysis of macrophages and promote LPS stimulated macrophage immune response (Leng et al., 2022). Many studies have found that Piezo1 not only exerts an influence on regulating macrophage function and polarization in response to various stimuli, such as inflammatory responses and mechanical stimuli (Tang et al., 2023), but is also widely involved in macrophage-mediated inflammatory diseases, such as pneumonia (Wang L. et al., 2020), atherosclerosis (Lin C. et al., 2022), atherosclerosis (Lin C. et al., 2022) and osteoarthritis (Evans et al., 2018; Jebari-Benslaiman et al., 2022). For B cells, their ability to process presenting antigens depends on Piezo1. Inhibiting Piezo1 with inhibitors reduces the ability of B cells to process presenting antigens other than soluble antigens, providing a possible direction for improving vaccine efficacy (Kwak et al., 2023). For microglia, Piezo1 is closely related to the immune response and migration of microglia, as confirmed by the use of Piezo1 inhibitors and activators in separate experiments (Zhu et al., 2023).

### 2.5 Other functions

For other organs, Piezo1 signals pulmonary vascular high permeability by promoting internalization and degradation of the endothelial adhesion junction protein VE-cadherin. Piezo1 was found to be related to the regenerative repair of the bladder, and the results suggest that Piezo1 can influence the proliferative ability (Wang X. et al., 2023). In addition, in primary cultured mouse bladder urothelial cells, the downregulation of Piezo1 affects the reception of mechanical stimulation of the bladder, and the high expression of Piezo1 in the bladder indirectly indicates the important role (Peyronnet et al., 2013). For the liver, Piezo1 alleviates acute liver injury caused by acetaminophen by activating nuclear factor erythroid 2-related factor 2 and reducing mitochondrial reactive oxygen species (Wang Q. et al., 2023). In addition, inducing and inhibiting ferroptosis makes a contribution to the treatment of drug-resistant tumors, ischemic organ damage, and other degenerative diseases related to overwhelming lipid peroxidation (Jiang X. et al., 2021; Hirata et al., 2023). For wound healing, the mechanical sensing mediated by Piezo1 drives the transformation of fat cells into fibroblasts that form scars, thereby promoting wound fibrosis. Inhibiting Piezo1, in turn, could target the transformation of fat cells into fibroblasts to prevent and reduce scarring (Griffin et al., 2023). Recent studies have also found that Piezo1 channels play an important role in pain perception. Mechanical stimulation can activate Piezo1 channels, which in turn cause the excitation of neurons and the transmission of pain signals. These findings provide new targets and strategies for pain treatment (Kim et al., 2012).

## 3 The signal pathways involved by Piezo1 in diseases

**FIGURE 3:**
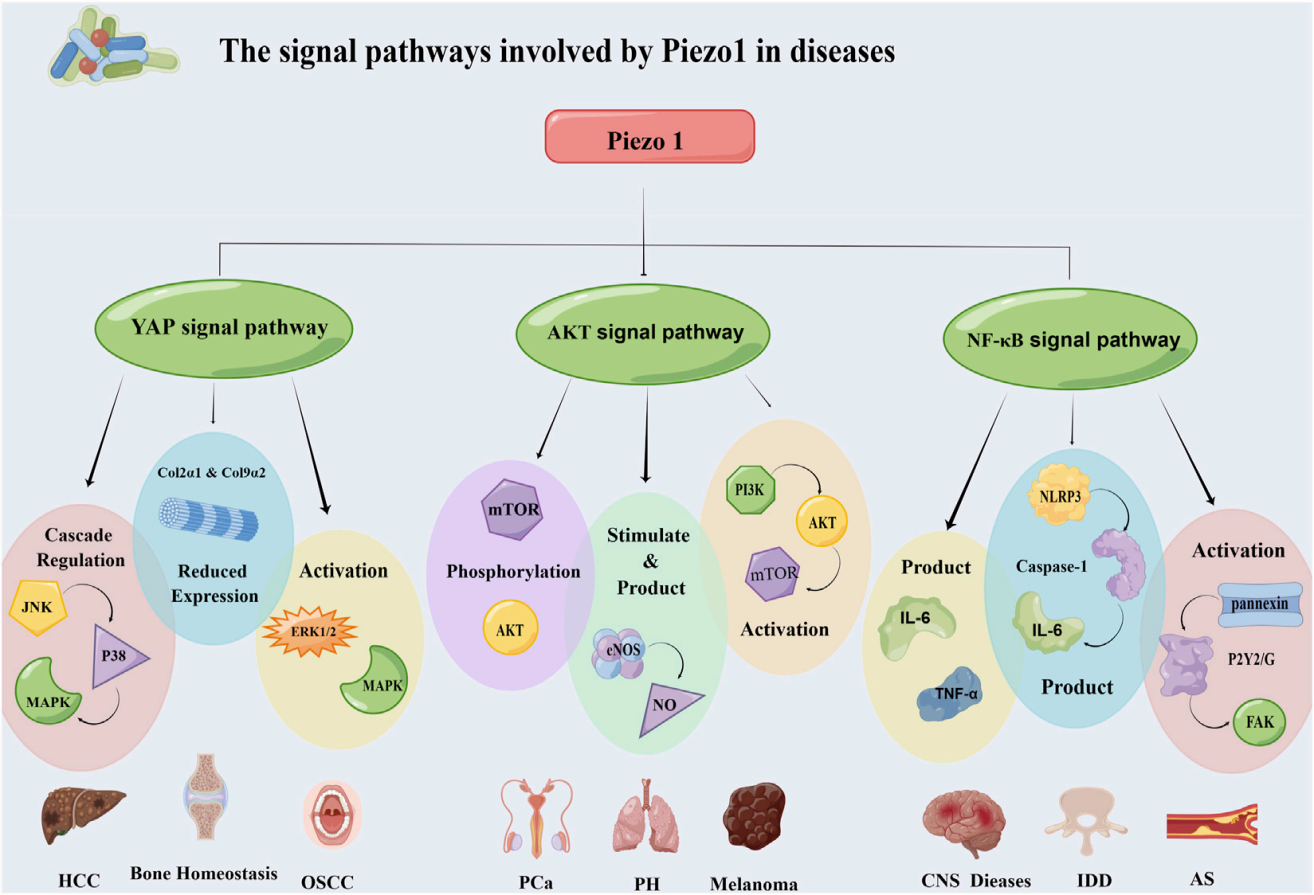
Piezo1 affects the occurrence and development of diseases through multiple signaling pathways. It mainly works through three pathways: YAP signaling pathway, Akt signaling pathway and NF-κB signaling pathway. The three pathways regulate the progression of hepatocellular carcinoma, oral squamous cell carcinoma, prostate cancer, atherosclerosis and other diseases through different mechanisms. Abbreviation: JNK: C-Jun N-terminal kinase; MAPK: Mitogen-activated protein kinases; ERK 1/2: Extracellular signal-regulated kinase 1/2; mTOR: Mammalian target of rapamycin; eNOS: Endothelial nitric oxide synthase; PI3K: Phosphatidylinositol 3-kinase; FAK: Focal adhesion kinase; NLRP3: NOD-like receptor thermal protein domain associated protein 3; HCC: Hepatocellular carcinoma; OSCC: Oral squamous cell carcinoma; PCa: Prostatic carcinoma; PH: Pulmonary hypertension; IDD: Intervertebral disc disease; AS: Atherosclerosis.

In human diseases, Piezo1 influences the development of disease through a variety of signaling pathways, including the FGF1/FGFR1 signaling pathway, YAP signaling pathway, BMP2/Smad signaling pathway, Akt signaling pathway, NF-κB signaling pathway, ROCK pathway, and JNK1/mTOR signaling pathway (Han et al., 2019; Wang S. et al., 2020; Hasegawa et al., 2021; Li J. et al., 2022; Malko et al., 2023). But Piezo1 mainly works in the following three signaling pathways (Figure 3).

### 3.1 YAP signaling pathway

The Hippo pathway is a classic tumor-associated highly conserved pathway. Transcriptional coregulatory factor YAP and transcriptional coactivator carrying PDZ-binding motifs (TAZ) are important effectors of the Hippo pathway (Zhao et al., 2008). YAP/TAZ is a sensor and mediator of mechanical signals in the cellular microenvironment (Panciera et al., 2017). Among them, YAP is an important mechanically sensitive transcription activator that can regulate cell proliferation and differentiation, closely related to the occurrence and development of different diseases (Halder et al., 2012). Piezo1 plays a specific role in regulating YAP translocation.

In hepatocellular carcinoma (HCC), the Ca^2+^ reaction induced by Piezo1 activation can promote YAP phosphorylation, which is mediated by JNK and P38 cascade regulation (Liu S. et al., 2021). Mitogen-activated protein kinases (MAPK) is a group of serine-threonine kinase. The MAPK signaling pathway can be activated through signal transduction of cell surface receptors (Delire and Stärkel, 2015), and improper regulation of this pathway leads to abnormal cell behaviors. After MAPK mediates the phosphorylation of YAP, activated YAP promotes cell proliferation, inhibits cell apoptosis, and ultimately promotes carcinogenesis (Liu S. et al., 2021). Piezo1 can also regulate bone homeostasis through the YAP pathway. Bone homeostasis depends on the overall balance between bone resorption and bone formation (Feng and McDonald, 2011). In addition, promoters of type II collagen-1 (Col2α1) and type IX collagen-2 (Col9α2) are known to be activated by YAP. In this case, Piezo1 acts as a mechanical regulator that directly senses the mechanical load and reduces YAP30 nuclear localization by turning down its expression (Zhang Y. et al., 2022). After the decrease of YAP nuclear localization, the expression of Col2α1 and Col9α2 is further decreased, which leads to the increase of osteoclast differentiation, affects the occurrence of osteoclasts, and thus interferes with bone home (Wang L. et al., 2020).

### 3.2 Akt signaling pathway

Akt is a serine/threonine kinase that is a central node in many signaling pathways (Revathidevi and Munirajan, 2019). The traditional pathway through which Akt exerts its primary carcinogenic role is the PI3K/Akt signaling pathway (Fresno Vara et al., 2004), with PI3K, Akt, and mTOR as the three major nodes (Kauffmann-Zeh et al., 1997). Overexpression and phosphorylated activation of Akt are two major events widely occurring in human tumors (Cerami et al., 2012) and may be involved in many aspects of tumor development, including proliferation (Zhou et al., 2001), apoptosis (Spokoini et al., 2010), tumor suppressor factor inhibition (Gomes et al., 2014), lipid metabolism (Liu et al., 2016), and angiogenesis (Semenza, 2002).

Prostate cancer is one of the most common cancers in men, and the expression level of the Piezo1 channel in human prostate cancer is significantly higher than that in non-malignant tissues. Moreover, there is experimental evidence that knocking out the Piezo1 channel can lead to reduced proliferation and migration of prostate cancer cells, indicating the anti-proliferative effect of Piezo1 knocking out. When Piezo1 is activated, calcium flow can be increased to further activate the Akt/mTOR signaling pathway, leading to Akt/mTOR phosphorylation, which promotes the proliferation and migration of prostate cancer cells and the growth of prostate tumors (Han et al., 2019).

Similarly, in the case of melanoma, Piezo1 promotes the malignant progression of the disease through Akt/mTOR signaling. Piezo1 ion channel protein can regulate calcium concentration in melanoma cells, further increasing the activation level of the PI3K/Akt/mTOR pathway, thereby regulating the malignant progression of melanoma (Zhang S. et al., 2022).

### 3.3 NF-κB signaling pathway

NF-κB is an important intracellular nuclear transcription factor (Oeckinghaus et al., 2011). Previous studies have speculated that it can promote tumor development through the following three pathways: Releasing reactive oxygen species leads to DNA damage and gene mutations (Huber et al., 2004; Liou and Storz, 2010); controlling Epithelial-Mesenchymal transition (EMT) and metastasis to promote cancer progression; upregulation of vascular endothelial growth factor and its receptors to control tumor angiogenesis (Xie et al., 2010). NF-κB serves as a key factor connecting immunity and inflammation (Gilmore, 2003), and Piezo1’s role in regulating inflammatory response through this pathway is cell-type specific.

In the occurrence and development of atherosclerosis, when endothelial cells are naturally exposed to interfering and atherogenic blood flow, they will activate endothelial Piezo1, leading to calcium ion influx, and then control the blood flow induced ATP release and subsequent activation of the P2Y2/G protein alpha subunits Galphaq and Galpha11 (Gq/G11) by activating pannexin channels, and then promote endothelial cell NF-κB activation through the mediation of integrin α5β1. This process requires phosphorylation of focal adhesion kinase downstream of integrin (Albarrán-Juárez et al., 2018b).

Intervertebral disc degeneration (IDD) is the main cause of back, neck, and nerve root pain (Risbud and Shapiro, 2014). Inflammatory factors produced by nucleus pulposus cells play an important role in the pathogenesis of IDD (Navone et al., 2017). Piezo1 activates the NOd-like receptor protein 3 (NLRP3) inflammasome in the nucleus pulposus via the Ca^2+^/NF-κB pathway. The NLRP3 inflammasome is a multiprotein cytoplasmic molecule comprising receptors, junctions, and effector molecules (Gross et al., 2011), and its abnormal activation leads to the occurrence of inflammatory diseases. Piezo1 induces initiation and assembly of the inflammasome via the Ca^2+^/NF-κB pathway, increasing mechanical-stretching-mediated caspase-1 activation in nucleus pulposus cells. Subsequently, caspase-1 converts pre-IL-1β to IL-1β, and accelerates the production and maturation of IL-1β, thereby leading to the production and development of IDD through pathways such as promoting extracellular matrix degradation, inducing inflammatory cascade reactions, promoting angiogenesis and new nerve innervation, and inducing apoptosis of nucleus pulposus cells (Sun Y. et al., 2020).

In the case of microglia, they are highly mobile under both physiological and pathological conditions and sense mechanical signals in the microenvironment (Ayata and Schaefer, 2020). Abnormal increase of pro-inflammatory signals from microglia is an important pathological factor in brain aging and the pathogenesis of central nervous system damage and neurodegenerative diseases (Bruno et al., 2021). The mechanosensitive Piezo1 channel is functionally expressed in microglia, and the Piezo1 channel activates and inhibits the NF-κB inflammatory signaling pathway by initiating intracellular Ca^2+^ signaling, thereby down-regulating the proinflammatory function of microglia, especially the production of TNF-α and IL-6, and then it can delay the development of brain aging, central nervous system damage, and neurodegenerative diseases (Malko et al., 2023).

In general, Piezo1 is involved in a variety of signaling pathways for disease progression, including the YAP, Akt, and NF-κB signaling pathways that promote the development of diseases, especially tumors. Exploring Piezo1’s involvement in disease signaling could lead to a clearer understanding of the related diseases and the search for new therapeutic targets. Most of what Piezo1 does in the NF-κB pathway is related to inflammation, and its role in tumors is not fully understood, which could be pursued as a line of inquiry.

## 4 Association of Piezo1 with tumors

Piezo1 is abnormally expressed in a variety of tumor tissues, and it is related to the malignant degree of some tumors and the survival rate of patients (Table 1). In addition, Piezo1 plays an important role in numerous biological processes in tumors (Table 2). Next, we will describe the specific mechanism by which Piezo1 regulates tumor characteristics in detail.

**TABLE 1:** The expression of Piezo1 in tumors and its association with tumor stage, grading and patient survival.

Tumors	Piezo1 expression	Association of higher Piezo1 expression with higher tumor stage and grading	Impact of higher Piezo1 expression on prognosis and survival rates	Ref.
Glioma	↑	Positive correlation	↓	Chen et al. (2018), Qu et al. (2020)
Oral squamous cell carcinoma	↑	-	-	Hasegawa et al. (2021)
Melanoma	↑	-	-	Zhang et al. (2022b)
Gastric cancer	↑	Positive correlation	↓	Zhang et al. (2018), Wang et al. (2021)
Prostate tumor	↑	-	-	Kim et al. (2022a)
Esophageal squamous cell carcinoma	↑	-	-	Gao et al. (2021)
Colon cancer	↑	Positive correlation	↓	Sun et al. (2020b), Li et al. (2023a)
Breast carcinoma	↑	-	↓	Li et al. (2015), Lin et al. (2022b)
Ovarian cancer	↑	-	-	Xiong et al. (2022)
Hepatocellular carcinoma	↑	Positive correlation	↓	Li et al. (2022d)
Pancreatic ductal adenocarcinoma	↑	-	-	Aykut et al. (2020)
Prostate cancer	↑	-	-	Han et al. (2019)
Pancreatic cancer	↑	Positive correlation	↓	Song et al. (2022)
Non-small cell lung cancer	↓	Negative correlation	↑	Huang et al. (2019)

**TABLE 2:** Treating tumors by acting on Piezo1-related mechanisms.

Tumors	Signaling molecules	Mechanism	Effects on tumors	Ref.
Human epidermoid carcinoma	Rac1	Piezo1 can block the activation of actin Rac1 by promoting Ca^2+^ influx and activating potassium channel KCa3.1	Piezo1 inhibits macropinocytosis	Kuriyama et al. (2022)
Glioma	FAK	Piezo1 localizes to focal adhesions and activates Integrin-FAK signaling	Tissue mechanics and Piezo1 regulate the malignant growth and invasion of glioma through a feedforward mechanism	Chen et al. (2018), Qu et al. (2020)
Oral squamous cell carcinoma	YAP	YAP regulates the Piezo1 signaling to participate in the activation of ERK1/2 and P38 MAPK	The high expression of Piezo1 promotes the proliferation of oral squamous cell carcinoma	Hasegawa et al. (2021)
Hepatocellular carcinoma	Rab5c	Recruitment and binding of Rab5c upregulates the TGF-β signaling pathway	Absence of Piezo1 Inhibit the proliferation, migration, invasion and EMT of hepatocellular carcinoma cells, as well as the growth and metastasis of tumors	Li et al. (2022e)
	TGF-β/Smad2/3	Piezo1 activates TGF-β/Smad2/3 signaling pathway by recruiting Rab5c	Piezo1 promotes EMT, tumor growth and progression	Li et al. (2022d)
	HIF-α	Integrin β1/Piezo1 activation/Ca^2+^ influx/HIF-1α ubiquitination/VEGF, CXCL16 and IGFBP2 pathways	Matrix stiffness drives angiogenesis in hepatocellular carcinoma through Integrin β1/Piezo1 activation/Ca^2+^ influx/HIF-1α ubiquitination/VEGF, CXCL16 and IGFBP2 pathways	Li et al. (2022c)
Melanoma	Akt/mTOR	Piezo1 activates the PI3K/AKT/mTOR signaling pathway	Piezo1 controls cell viability, metastasis, invasion, and transepithelial migration by activating AKT phosphorylation	Zhang et al. (2022b)
Stomach cancer	TFF1	Piezo1 promotes cell migration by interacting with TFF1	Piezo1 deletion causes drastic changes in the shape of cancer cells, limiting movement, and blocking the cell cycle	Zhang et al. (2018)
	HIF-α/VEGF/Calpain1/2	Piezo1 induces Calpain1/2 expression by up-regulating HIF-α and VEGF	Piezo1 regulates proliferation, angiogenesis, metastasis and EMT in gastric cancer tissue	Wang et al. (2021)
Prostate tumor	YAP	YAP is activated via the Piezo1-Src-YAP axis	Piezo1 promotes tumor migration by activating YAP to advance the cell cycle	Kim et al. (2022a)
Esophageal squamous cell carcinoma	P53	The downregulation of Piezo1 upregulated the expression of P53 and its downstream factors Bax and Caspase 3	Piezo1 downregulation arrests cell cycle, induces apoptosis, and inhibits invasion and metastasis through Piezo1-P53-Bax-Caspase 3 axis	Gao et al. (2021)
	Bax			
	Caspase			
Colon cancer	HIF-1α/VEGF	Piezo1 regulates tumor metastasis through Piezo1-MCU-HIF-1α-VEGF pathway	Piezo1 promotes metastasis and inhibits healing	Sun et al. (2020b)
	NFAT1	Piezo1 knockdown inhibits the stemness of CCSCs by inhibiting Ca^2+^/NFAT1 signaling and deregulation of NFAT1	Piezo1 maintains the stemness of colon cancer stem-like cells	Li et al. (2023a)
Breast cancer	ERM	Piezo1 activation inhibits thrombin induced phosphorylation of ERM	Piezo1 is an inhibitor of thrombinase-induced blistering in breast cancer cells	O’Callaghan et al. (2022)
Ovarian cancer	YAP	Activation of Hippo/YAP signaling	Piezo1 promotes ovarian cancer metastasis	Xiong et al. (2022)
Pancreatic ductal adenocarcinoma	H+	Excessive acidification attenuates Piezo1 activity	In acidosis environments, Piezo1 activity weakens and its response to high pressure weakens, promoting the migration of pancreatic stellate cells	Kuntze et al. (2020)
	MYL9	Piezo1 enhances MYL9 phosphorylation through Ca^2+^ influx		
	Rb1	Piezo1 inhibits Rb1 expression by regulating histone decarboxylase 2	Elimination of Piezo1 in myeloid cells could prevent cancer	Aykut et al. (2020)

Abbreviation: YAP: Yes-associated protein; Rac1: Ras-related C3 botulinum toxin substrate 1; FAK: focal adhesion kinase; ERK1/2: Extracellular signal-regulated kinase 1/2; MAPK: Mitogen-activated protein kinases; TGF-β: Transforming growth factor-β; HIF-α: Hypoxia-inducible factor-α; VEGF: vascular endothelial growth factor; CXCL16: CXC, chemokine ligand 16; IGFBP2: Insulin-like growth factor binding protein 2; mTOR: mammalian target of rapamycin; PI3K: Phosphatidylinositol 3-kinase; TFF1: Trefoil factor family 1; EMT: Epithelial-Mesenchymal Transition; Bax: Bcl-2-associated X protein; Caspase: Cysteinyl aspartate specific proteinase; NFAT: Nuclear factor of activated T cells; ERM: ezrin, radixin and moesinn; MYL9: Myosin Light Chain 9; Rb1: Retinoblastomal; CCSCs: colon cancer stem cell-like cells.

### 4.1 Cell metabolic reprogramming

Tumor cells are often in a state of nutrient deficiency due to their continuous proliferation or special location of growth. To adapt to this extreme environment, tumor cells often reprogram their cellular metabolism to take up essential nutrients from the nutritionally deprived environment and adjust intracellular metabolic pathways to supply the more important biological processes (Pavlova Natalya and Thompson, 2016). Macropinocytosis is an important non-selective extracellular amino acid uptake pathway, which has been reported in tumor cells (Del Bello et al., 2022; Xia et al., 2023) and even tumor stromal cells (Zhang et al., 2021). Macropinocytosis is dependent on the formation of peripheral folds, whose formation and migration are regulated by actin (Isogai et al., 2015). Activated Piezo1 can block the activation of actin Rac1 by promoting Ca^2+^ influx and activating potassium channel KCa3.1, thereby inhibiting macropinocytosis in human epidermoid carcinoma (Kuriyama et al., 2022). The Piezo1 activator Yoda1 inhibits macropinocytosis by interfering with the physiological formation of peripheral folds, and Yoda1 specifically activates the Piezo1 gene without any effect on other endocytic pathways, making it a potential therapeutic option for tumor inhibition by targeting amino acid metabolism.

In addition, purine metabolism, which is inseparable from cell proliferation, was previously found to be enriched in several cancers (Chen X-H. et al., 2019; Chen S. et al., 2022; Liu et al., 2023) and associated with poor efficacy of immunotherapy (Li S. et al., 2022). Through pathway enrichment analysis and correlation detection using KEGG and Wiki databases, it was found that Piezo1 potentially facilitates the growth of breast cancer by modulating the purine metabolism of GUK1, POLD1 and APRT (Chen and Chen, 2022).

### 4.2 Proliferation and apoptosis

Tumor cells relieve the regulation of proliferation signals and achieve sustained proliferation, which is the most fundamental characteristic of tumor cells (Hanahan and Weinberg, 2011). The effect of cell proliferation signal may be related to Piezo1 on the cell membrane. For example, in glioma, tumor cells specifically rely on Piezo1 for growth and proliferation, while Piezo1 is not expressed in normal glial cells (Chen et al., 2018). Moreover, knocking out Piezo1 gene has no significant effect on the volume and mitosis of normal glial cells (Chen et al., 2018). In addition, Hasegawa et al. also found that YAP regulates Piezo1 to participate in the proliferation and growth of oral squamous cell carcinoma (OSCC) (Hasegawa et al., 2021). After knocking down Piezo1 gene or using Piezo1 inhibitor GsMTx-4, the proportion of OSCCs and cell proliferation ability decreased (Hasegawa et al., 2021). Among them, Piezo1 is also involved in the activation of agonist-dependent ERK1/2 and p38 MAPK (Hasegawa et al., 2021). Given that ERK1/2 and p38 MAPK are involved in cell proliferation (Nishimoto and Nishida, 2006; Zhang et al., 2019), their activation by Piezo1 may be one of the mechanisms promoting tumor cell proliferation. Besides, fluid shear stress and Yoda1 synergistically activate Piezo1 in prostate cancer cells by increasing the proportion of early S/S/G2/M phase in the cell cycle through the activation of YAP/TAZ, while transplanted Piezo1-silenced prostate cancer cells failed to proliferate *in vivo* (Kim O-H. et al., 2022). However, the signaling pathway between Piezo1 and YAP/TAZ complex is still unclear, and whether there is a synergistic effect between them in the process of tumor cell proliferation is still a question that needs to be further explored.

Unlimited proliferation of tumor cells depends on continuous cell division in addition to receiving proliferative signals, which is closely linked to cell cycle surveillance. Alterations in Piezo1 expression have been observed in several tumors by regulating key cell cycle regulators and thus affecting tumor cell proliferation. In melanoma cells, knockdown of Piezo1 resulted in alterations in cell cycle-related genes, with a significant decrease in the expression of key effectors CDK2 and CyclinD1 genes and an increase in the expression of classical tumor suppressors P21 and PTEN (Zhang S. et al., 2022). In addition, Piezo1 knockdown induced G1 phase block in gastric cancer cells and downregulated phosphorylated Rb, Cyclin D1, CDK4, and CDK6 (Zhang et al., 2018), suggesting that Piezo1 is required for gastric cancer cell proliferation. Similarly, a study on prostate cancer showed that Piezo1 downregulation inhibited the expression of Cyclin D1 and CDK4, hindering the assembly of the Cyclin D1-CDK4 complex and thus impeding cell cycle progression in prostate cancer cells (Han et al., 2019). Interestingly, the present study demonstrated that Piezo1 regulation of the cell cycle in prostate cancer cells is associated with the Akt/mTOR pathway, but not with the ERK1/2 pathway (Han et al., 2019).

In addition to uncontrolled cell proliferation, inhibition of apoptosis is also a typical feature of tumor cells (Hanahan and Weinberg, 2011). Various key events of apoptosis are concentrated in mitochondria (Green and Reed, 1998). Piezo1 interferes with the apoptosis of tumor cells by inducing Ca^2+^ influx and mediating mitochondrial dysfunction. In the ultrasonic-targeted microbubble therapy of pancreatic ductal cell carcinoma, Piezo1 blockade reduced Ca^2+^ influx, decreased cytochrome C and Bax, and increased Bcl-2, which finally assisted microbubble to induce apoptosis of cancer cells (Song et al., 2022). Moreover, the activation of Piezo1 in prostate cancer amplifies the death-induced signal on the apoptosis pathway mediated by TNF-related apoptosis-inducing ligand (TRAIL) via Ca^2+^, leading to mitochondrial outer membrane permeability and mitochondrial dysfunction, and improving the therapeutic effect of TRAIL through the intrinsic apoptosis pathway (Hope et al., 2019). In addition to Ca^2+^-related apoptotic pathways, Piezo1 also interacts with P53 in esophageal squamous cell carcinoma cells (ESCCs) (Gao et al., 2021). The expression of P53 downstream factors Bax and Caspase protein was significantly upregulated when Piezo1 was downregulated, and the mouse experiment shows that the growth rate of ESCCs was decreased in association with the upregulation of these factors (Gao et al., 2021). These suggest that Piezo1 downregulation induces ESCCs apoptosis through Piezo1-P53-Bax-Caspase 3 axis.

In summary, Piezo1 has an ingenious role in the activation of tumor cell proliferation signals and cell cycle operation, and has been implicated in multiple tumor apoptotic pathways, which is why the effect of Piezo1 on tumor proliferation and apoptosis is so complex.

### 4.3 Stem cells

Cancer stem cells (CSCs) are a population of cells with the ability to differentiation, proliferation, and self-refinement, which play an important role in tumor progression (Chiba et al., 2007), inflammation (Zhao et al., 2023), drug resistance (Choi et al., 2020), and prognosis (Xu et al., 2023). Abnormal mechanistic signals in TME can affect the proliferation, differentiation, stemness, and other biological behaviors of CSCs (Shen et al., 2021; Nguyen Ho-Bouldoires et al., 2022). Therefore, the regulatory mechanisms acting on the mechanical microenvironment of CSCs have great potential for clinical applications (Tian et al., 2021), and Piezo1 is just related to the maintenance of stemness of CSCs. Compared with low stemness colon cancer stem cell-like cells (CCSCs) and non-CCSCs, Piezo1 is highly expressed in CCSCs with high stemness, and the population with high Piezo1 expression is associated with clinical stage (Li R. et al., 2023). Piezo1 can maintain the stemness of CCSCs through Ca^2+^/NFAT1 signaling pathway, and the stemness and tumorigenic ability of CCSCs are impaired when Piezo1 is knocked down, while the cloning potential of CCSCs is increased when Piezo1 is overexpressed (Li R. et al., 2023). Furthermore, Piezo1 also affected the stemness and *in vivo* growth of glioblastoma stem cells (GSCs) (Chen et al., 2018).

In addition to CSCs, the effect of Piezo1 on another pluripotent stem cell, mesenchymal stem cell (MSC), is intriguing. In human dental pulp-derived MSCs, Piezo1 activation stimulates MSC migration by inducing ATP release and activation of the P2 receptor purinergic signaling pathway as well as the downstream PYK2 and MEK/ERK signaling pathways (Mousawi et al., 2020). MSCs are homing to tumor tissues under the regulation of chemokines (Chamberlain et al., 2007), and will transform into tumor-associated fibroblasts upon stimulation by tumor cells (Spaeth et al., 2009). Due to the high affinity of MSCs to tumor cells, an increasing number of studies have used MSCs as a delivery carrier of anti-tumor drugs (Fan et al., 2023). Therefore, the effect of Piezo1 on the homing efficiency of MSCs and tumor progression is an interesting direction to explore.

### 4.4 Angiogenesis

While normal vasculature is in a quiescent state and only activates transiently in response to injury and certain physiological conditions, there are “angiogenic switches” in tumor cells that continuously activate the vasculature to generate new blood vessels (Hanahan and Weinberg, 2011). The angiogenic switch is regulated by both pro-angiogenic and anti-angiogenic factors, and Piezo1 promotes tumor angiogenesis by up-regulating pro-angiogenic factors and their upstream and downstream effectors (Han et al., 2019). In HCC, the activation and upregulation of Piezo1 inhibit hypoxia-inducible factor 1 (HIF-1) ubiquitination, which in turn enhances the expression and secretion of downstream pro-angiogenic factors such as vascular endothelial growth factor (VEGF), CXC chemokine ligand 16 (CXCL16) and insulin-like growth factor binding protein 2 (IGFBP2), ultimately accelerating HCC angiogenesis (Li M. et al., 2022). Moreover, in peritoneal metastatic tumor tissues of gastric cancer, Piezo1 also regulates the expression of downstream molecules Calpain1/2 by up-regulating HIF-1 and VEGF, promoting tumor angiogenesis and metastasis (Wang et al., 2021). Among them, Calpain1/2 promote angiogenesis and endothelial motility in response to VEGF signaling through the Ezrin/Calpain/PI3K/AMPK/eNOS signaling axis (Youn et al., 2009). Furthermore, Piezo1 activation in periosteal stem cells also upregulates angiogenic factors such as bone morphogenetic protein-2 (BMP-2) and VEGF-A through the YAP/β-catenin signaling pathway (Liu et al., 2022); however, whether this Piezo1/YAP/β-catenin signaling axis also exists in tumor cells still needs further investigation.

### 4.5 Invasion and metastasis

**FIGURE 4:**
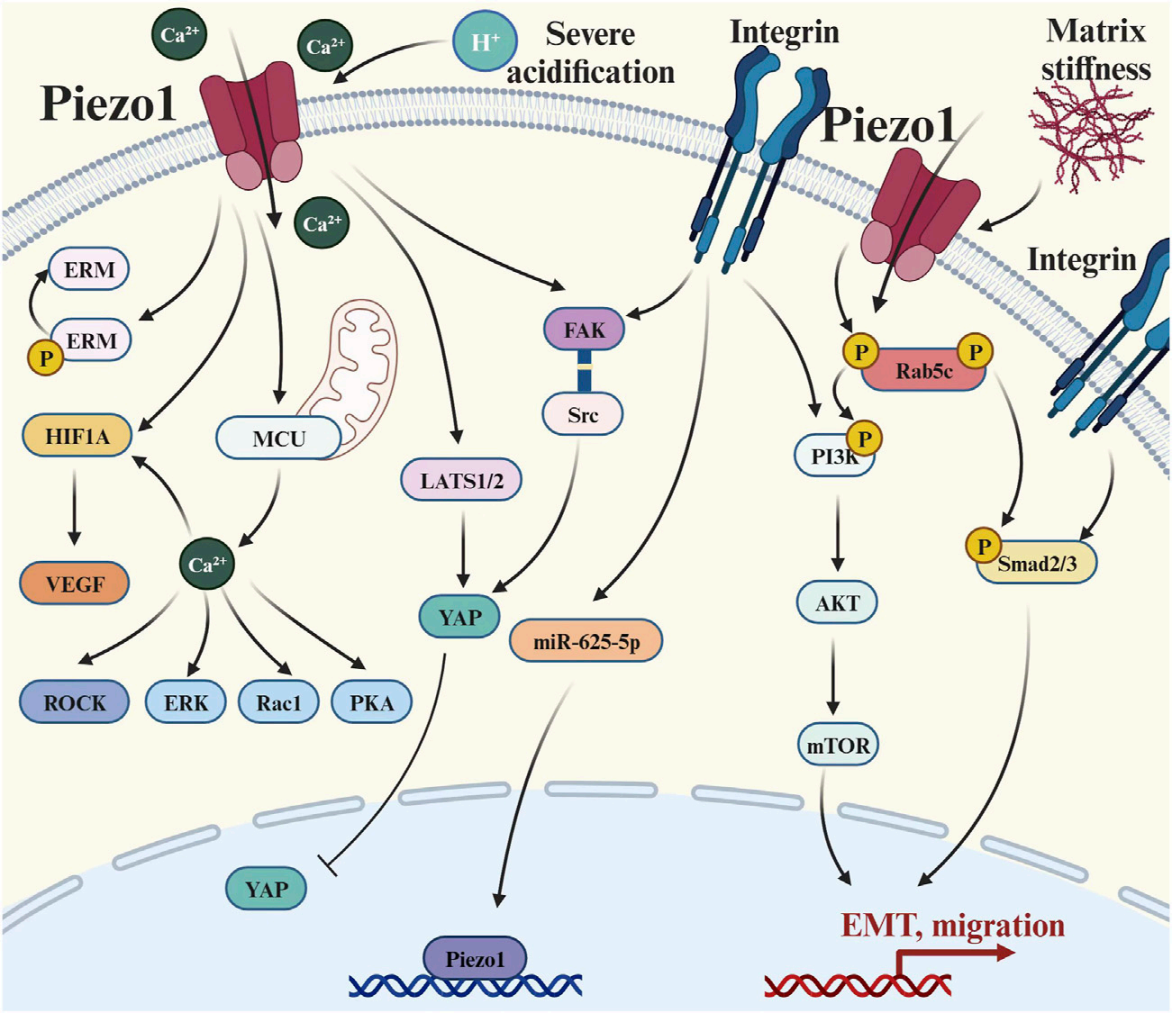
Piezo1 crosstalks with multiple signaling pathways to regulate tumor metastasis: (1) Extracellular matrix signal transduction: there is a positive feedback pathway between Piezo1 and matrix hardness, and also its activity is affected by environmental pH; (2) Integrin-mediated pathways: such as Integrin-Adhesion Spot, Classical Smad2/3 Pathway, and Non-Smad2/3 pathway; (3) Cytoskeletal-related pathways: Causing changes in a series of actin skeleton-related effectors such as ERM, ROCK, and Rac1; (4) Hippo pathways: the LATS1/2-YAP axis and the Piezo1-Src-YAP axis; and (5) Ca^2+^-mediated pathways: elevated concentrations of Ca^2+^ caused upregulation of HIF-α and VEGF. Abbreviation: EMT, Epithelial-mesenchymal transition; ERM, Ezrin, radixin and moesinn; ERK1/2, Extracellular signal-regulated kinase 1/2; FAK, Focal adhesion kinase; HIF-1, Hypoxia-inducible factor 1; LATS1/2, Large tumor suppressor 1/2; MCU, Mitochondrial calcium uniporter; mTOR, Mammalian target of rapamycin; P, Phosphorylation; PI3K, Phosphatidylinositol 3-kinase; PKA, Protein kinase A; Rac1, Ras-related C3 botulinum toxin substrate 1; ROCK, Rho-associated protein kinase; Smad2/3, SMAD Family Member 2/3; VEGF, Vascular endothelial growth factor; YAP, Yes-associated protein.

Tumor metastasis is a multi-step cascade process, which can be roughly divided into local invasion of tumors, internal infiltration into the peripheral vasculature and lymphatics, survival in the circulatory system and metastasis, extravasation from the vascular lumen into the parenchymal tissues, and finally adaptation to the microenvironment of the metastasis to complete colonization (Talmadge and Fidler, 2010). A large body of literature shows that Piezo1 plays an important role in tumor migration (Li et al., 2015), extravasation (Zhang S. et al., 2022), and distant metastasis (Lin CY. et al., 2022), and promotes local invasion and distant metastasis of tumors (Figure 4). The Piezo1-HIF-1α-VEGF pathway (Sun YH. et al., 2020; Wang et al., 2021), Piezo1-Src-YAP axis (Kim O-H. et al., 2022), and Piezo1-LAST1/2-YAP axis (Xiong et al., 2022) are all potential mechanisms of tumor metastasis. However, some studies have also proposed opposite conclusions. For example, in human non-small cell lung cancer, especially lung adenocarcinoma patients, high Piezo1 expression correlates with better overall survival, and knockdown of Piezo1 significantly promotes cancer cell migration *in vitro* and tumor growth *in vivo* (Huang et al., 2019).

In order to better adapt to the complex and variable microenvironment during metastasis, metastatic tumors often differ from primary tumors in biological and behavioral characteristics, especially the invasive changes induced by EMT (Park et al., 2019; Mao et al., 2020). Several studies have shown that Piezo1 can help tumor cell metastasis by promoting EMT (Gao et al., 2021; Wang et al., 2021). Piezo1 induces EMT by activating the TGF-β/Smad2/3 signaling pathway through the recruitment of Rab5c (Li et al., 2022d). Knockdown of Piezo1 transformes the HCCs from a spindle-like literal morphology to a cobblestone-like epithelial morphology, with a decreased ability of cells to migrate, and the growth of subcutaneous and orthotopic xenograft tumors was also inhibited (Li et al., 2022d). Moreover, Piezo1 also promotes tumor metastasis by activating the non-Smad pathway of TGF-β. Among them, PI3K/AKT/mTOR pathway regulates the invasion and metastasis process of melanoma upon activation by Piezo1, and the expression of EMT-related genes and invasion and metastasis-related genes (MMP2 and MMP9) in cells changes with the alteration of Piezo1 expression (Zhang S. et al., 2022). Similarly, in vitro experiments, Piezo1 downregulation induced reduced migration of prostate cancer cells by inhibiting Akt and mTOR phosphorylation (Han et al., 2019). In addition, Piezo1 has been implicated in apoptosis and senescence resistance of metastatic tumors. A study on colorectal cancer cells showed that compared with metastatic tumors, primary tumors had higher Pizeo1 expression and were more sensitive to TRAIL-mediated apoptosis induced by both mechanical force and shear stress (Greenlee et al., 2022).

During tumor metastasis, tumor cells generally undergo elongated-mesenchymal migratory movements, which are closely related to the integrin-FAK pathway (Wang CH. et al., 2020). The expression of Piezo1 was higher in high invasive gliomas than in low invasive gliomas, and its immunofluorescence images showed that Piezo1 was distributed at the adhesion spots mediated by integrins and FAK activation, indicating that Piezo1 may be related to the integrin-adhesion spot pathway and thus participate in the adhesion and migration of tumors (Chen et al., 2018). However, when integrin function is diminished, tumor cells will prefer non-adhesion dependent round-amoeboid migration (Carragher et al., 2006). Although the loss of Piezo1 in small cell lung cancer epithelial cells reduces the integrin affinity and cell adhesion, it simultaneously biases tumor cell migration toward the amoeboid mode, which moves in a more globular morphology depending on actin cytoskeleton rearrangement (McHugh et al., 2012). Both mesenchymal and amoeboid migration are closely related to the actin cytoskeleton (Ruiz-Lafuente et al., 2021). Yoda1-induced Piezo1 activation increases intracellular calcium levels through extracellular calcium inward flow and release of calcium from endoplasmic reticulum storage, and upregulates the activity of a series of downstream effectors including PKA, ERK, Rac1 and ROCK, leading to cytosolic ruffling and thus promoting cancer cell migration (Kim HS. et al., 2022). In addition, Piezo1 knockdown in gastric cancer tissues leads to GTP-Rac1 hyperactivation thereby interrupting the GTPGDP conversion cycle restriction of Rho protein, which finally causes drastic changes in the shape of the cancer cells and restriction of motility through reduction of stress fibers (Zhang et al., 2018). Thus, Piezo1 is essential for actin cytoskeleton-dependent migratory motility.

Piezo1 is not only activated by forces generated by cytoskeletal alterations, but also transduces extracellular mechanical signals. Piezo1 can sense the change in ECM stiffness and regulate the content of ECM markers by regulating calcium flux, which plays an important role in prostate cancer cell metastasis and extravasation (Lopez-Cavestany et al., 2023). In addition to matrix stiffness, environmental pH is also involved in the interaction between Piezo1 and the matrix, making the microenvironmental signal response in the TME more complex. Despite the severe fibrosis and high tissue pressure in PDACs, the extremely acidic environment inhibits Piezo1-mediated Ca^2+^ influx and prevents it from fully functioning. Therefore, pancreatic ductal cell carcinoma cells are weakly responsive to extremely high mechanical pressure and can survive under high pressure (Kuntze et al., 2020). When the tumor invades into a well perfused area, Piezo1 reactivates due to the loss of the acidified environment (Kuntze et al., 2020).

### 4.6 Tumor immune microenvironment

Although the continuous action of the immune system certainly eliminates a certain number of tumor cells, tumors gradually develop an immunosuppressive state through reprogramming the tumor immune microenvironment (TIME) (Lv et al., 2022). Immune cells are one of the main components of TIME, which are composed of monocyte-macrophages, T cells, B cells, and so on. Among them, tumor-associated macrophages (TAMs) are the most abundant tumor-infiltrating immune cells in the TME, which have a considerable role in tumor progression (Chen Y. et al., 2019). According to the binary polarization model, macrophages are functionally classified into M1 and M2 polarization states (Mills et al., 2000), especially M2 TAMs are strongly associated with tumor invasiveness (Li ZH. et al., 2023; Zhang XX. et al., 2023). Therefore, blocking the polarization of macrophages to M2 and promoting macrophage repolarization from M2 to M1 are very promising strategies for tumor therapy (van Dalen et al., 2018; Pan et al., 2020). Activation of Piezo1 in macrophages promotes IFN-γ and LPS-induced inflammation by activating NF-κB, and inhibition of STAT6 inhibits healing responses induced by IL-3 and IL-4 (Atcha et al., 2021). In other words, the expression of Piezo1 steered macrophages toward the pro-inflammatory M1 polarization state and away from the anti-inflammatory and pro-cancer M2 polarization state. Specifically, there may be a potential positive feedback link between Piezo1 and actin, which together promote the polarization of macrophages to M1 type and ultimately contribute to tumor therapy (Atcha et al., 2021). Moreover, it has also been shown that SDT-activated Piezo1 combined with ultrasound not only recruited macrophages in the orthotopically transplanted glioma model, but also increased the proportion of M1 macrophages (Chen L. et al., 2022).

In addition to macrophages, Tregs that play an immunosuppressive role in TIME also have a high degree of plasticity (Liu XJ. et al., 2021) and can intertransform with other regulatory T cells (Hinshaw et al., 2023; Tuomela and Levings, 2023). Piezo1 appears to regulate the differentiation of CD4^+^ cells in the direction of departing Tregs. Piezo1-deficient DCs directly regulate the secretion of the polarizing cytokines TGF- β1 and Interleukin-12 (IL-12), resulting in elevated TGF-βR2/Smad3 activity and reduced IL-12Rβ2/STAT4 activity, while inducing Tregs and Th1 cells to differentiate from each other (Wang et al., 2022). Furthermore, Piezo1 integrates the SIRT1/HIF1α-dependent metabolic pathway and the calcium-calcineurin phosphatase-NFAT signaling pathway, which also shifts the balance of Th1 cells and Tregs differentiation through IL-12 and TGF- β1 (Wang et al., 2022). Additionally, it has been corroborated that Piezo1 selectively inhibits the induced expansion of Tregs by attenuating TGF-β signaling, thereby inhibiting the balance of functional T cells to transfer to Treg cells (Jairaman et al., 2021).

Moreover, Piezo1 can inhibit the recruitment and expansion of myeloid derived suppressor cells (MDSCs), a heterogeneous cell population with immunosuppressive function and significantly associated with poor cancer prognosis (Bae et al., 2021; Jiang et al., 2023). Mechanical signaling stimulation promotes the recruitment and amplification of MDSCs by Piezo1 through inhibition of Rb1, and reduces CD4^+^ and CD8^+^ activation (Aykut et al., 2020). In pancreatic ductal adenocarcinoma, the Piezo1-deficient group had slower tumor growth and reduced MDSCs (Aykut et al., 2020). Piezo1 can inhibit the recruitment and amplification of MDSCs to suppress tumor development.

## 5 Piezo1 as a target for tumor therapy

**FIGURE 5:**
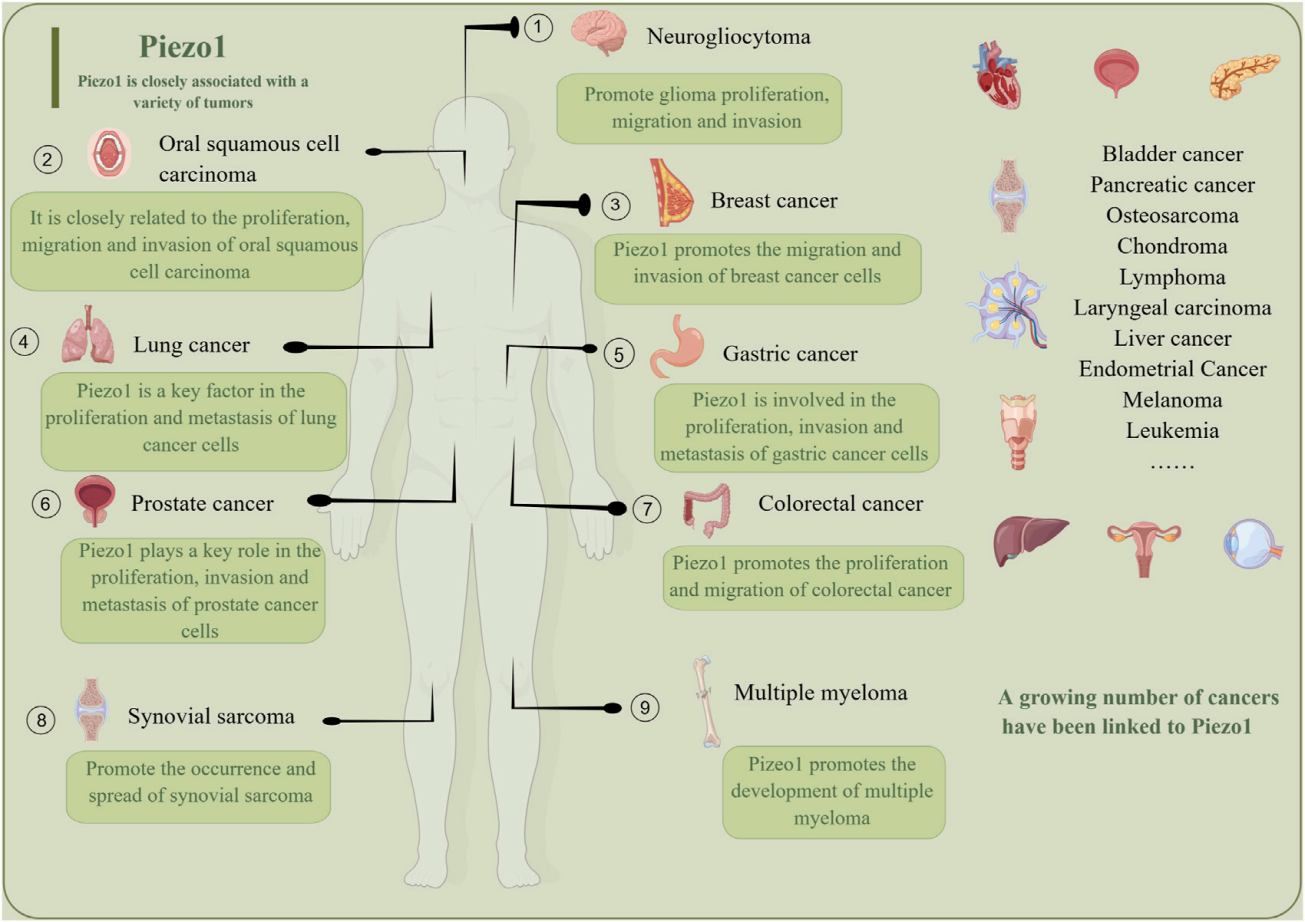
Piezo1 is a promising tumor treatment target, and a growing number of tumors are being found to be closely related to Piezo1. Piezo1 promotes the growth, migration, inhibition of apoptosis and proliferation of different tumors.

A growing body of research suggests that Piezo1 has a potentially important role in cancer therapy (Figure 5). Several studies have suggested that Piezo1 could be a potential therapeutic target for tumors. For example, in stomach cancer, Piezo1 works as a potential oncogene by promoting the proliferation and migration of cells involved in gastric cancer (Zhang et al., 2018). Similarly, Piezo1 acts as an oncogene in pancreatic cancer progression, and its activation regulates the cancer-tumor microenvironment interaction to accelerate the growth of pancreatic cancer tumors (Zhu et al., 2022). Additionally, Piezo1 may influence the metastasis and prognosis of colon cancer via the Piezo1-MCU-HIF-1α-VEGF axis (Sun YH. et al., 2020). For invasive human glioma, Piezo1 is overexpressed to activate integrin FAK signaling and promote tumor invasiveness. Interestingly, mutations in Piezos are associated with various human genetic diseases, confirming their potential as therapeutic targets (Zhao et al., 2019). However, both research on Piezo1 as a therapeutic target and its related drugs are in the early stages and require further exploration and validation. The following are the relevant drugs for Piezo1 as a therapeutic target.

### 5.1 Piezo1 inhibitors

In developing drugs related to Piezo1, there have been no reported clinical trials of Piezo1-specific inhibitors. Currently, some mainstream Piezo1 inhibitors do not directly inhibit the expression and/or activity of Piezo1, and their inhibitory effects are not specific (Zhao F. et al., 2022). For example, eicosapentaenoic acid can reduce the membrane stiffness and bending stiffness, indirectly reducing the deactivation time constant of Piezo1, thereby disrupting its function (Romero et al., 2019). The application of Piezo1 inhibitors in pancreatic acinar cells has been found to reduce the damage caused by pancreatic duct hypertension (Romac et al., 2018). Some new inhibitors are also being explored. Salvianolic acid B can inhibit Yoda1 and mechanically activated current, prevent aortic ring relaxation, and reverse the formation of foam cells caused by Yoda1. Salvianolic acid B also inhibits the formation of atherosclerotic plaque (Pan et al., 2022). In addition, the downregulation of Piezo1 induces apoptosis of ESCCs through the 3-axis of Piezo1-P53-BAx-caspase, and the downregulation of Piezo1 inhibits the development (Gao et al., 2021), perhaps this can become a new pathway for the application of Piezo1 inhibitors in tumor treatment.

Some studies have also found that drugs already in clinical use or small naturally occurring molecules may have the potential to suppress Piezo1. For example, studies have found that peptide inhibitors play an important role in preventing tumors and septic shock by comprehensively inhibiting Piezo1 (Aykut et al., 2020). In addition, MMAE is a synthetic anti-tumor drug associated with monoclonal antibodies. There is evidence to suggest that the combination of Piezo1 antibody and MMAE can not only promote tumor cell cycle arrest and apoptosis, but also slow down the development of ESCC xenograft tumor models without any side effects (Qin et al., 2022). In addition, genetic disruption of the neutral sphingomyelin inhibitor and sphingomyelin phosphodiesterase 3 can lead to the inactivation of Piezo1 (Shi et al., 2020), this may provide a new approach for the development of Piezo1 inhibitors.

### 5.2 Piezo1 agonist

Similarly, Piezo1 agonists are being used in basic experimental research. In contrast to inhibitors, today’s mainstream Piezo 1 agonists are all specific. Among them, the most discussed is the Yoda1 agonist. Studies have shown that the main and most stable ligand of Yoda1 is L13, which, when combined with it, can increase the relative flexibility of the peripheral arm region relative to the central pore cap region, thereby increasing mechanically induced protein movement (Botello-Smith et al., 2019). Multiple studies have provided ideas for activating Piezo1 through Yoda1 agonists and participating in tumor treatment. A study indirectly confirmed that Piezo1 can be activated by Yoda1 agonists under static conditions, and activation can increase the sensitivity of TRAIL (Knoblauch et al., 2023). Among them, TRAIL can specifically induce tumor cell apoptosis, but does not affect normal cells, making it one of the hotspots in tumor treatment (Hope et al., 2019). In addition, in the case of Piezo1, the agonist Yoda1, was found to effectively inhibit megalocytogenesis induced by epidermal growth factor (Kuriyama et al., 2022). Among them, megalocytosis promotes tumor cell survival through the non-selective uptake of extracellular proteins and nutrients (Song et al., 2020). In addition, research has shown that increasing the opening of Piezo1 cation channels can promote ferroptosis, which may become the direction of action for Piezo1 agonists in the future (Hirata et al., 2023).

### 5.3 Combination therapy

In addition, Piezo1 has been found to work in other tumor treatments or in combination with other anti-tumor drugs. A combination of microtubule depolymerizer and ultrasound therapy has been found to promote apoptosis in tumor cells. Among them, the ultrasound can destroy the microtubules of tumor cells by activating calpsin 1 and 2, thus enhancing the killing effect of microtubule depolymerizer. The increased entry of calcium ions after ultrasound is associated with the neighborhood around the tumor cells, and the location of Piezo1 near the tumor cells suggests that Piezo1, by activating the channel, facilitates the entry of calcium ions and thus enhances the therapeutic effect (Singh et al., 2021). In addition, several studies have raised the prospect of Piezo1 being used in combination with cytoskeletal drugs. The studies suggest that mitochondrial dysfunction, activated by Piezo1 through the ER-mitochondrial stress pathway, is associated with apoptosis (Tijore et al., 2022). Drugs that target the cytoskeleton, meanwhile, are used clinically as chemotherapeutic agents (Kubiak et al., 2020). The combination of the two related drugs could open up new avenues of tumor treatment in the future.

Overall, Piezo1 has a potentially important role as a target for tumor treatment. Although the development of drugs related to Piezo1 is still in its early stages, some progress has been made. These studies provide a strong basis for developing drugs related to Piezo1. However several studies contradict these conclusions. Unlike previous results, some studies suggest that lowering Piezo1 may promote the invasion and metastasis of lung cancer (McHugh et al., 2012). As a result, further research and clinical trials are needed that will help verify the efficacy and safety of Piezo1 as a tumor treatment target, and provide additional options for developing new treatment strategies.

A review of the recent literature on targeted drug design leads us to the following conclusions. Designing drugs that target Piezo1 has faced numerous difficulties. For one thing, the protein structure of Piezo1 is so complex and delicate that it has not yet been fully explored, making designing drugs to target a big challenge. Second, the molecular mechanism and therapeutic potential of Piezo1 have not yet been validated by suitable clinical compounds, which also limits progress in drug design. In addition, the universal nature of Piez1’s biological function makes specific drug use difficult. This requires a thorough understanding of the specific biological function of Piezo1 and the development of potential pharmacokinetics, or antagonists, with good drug-like properties. In summary, the drug design of Piezo1 faces several challenges and limitations that will require further research and efforts to address (Völkner et al., 2022).

## 6 Discussion and perspectives

Piezo1, as a mechanosensitive ion channel, is widely found in nonsensory tissues, especially in the lungs, bladder and skin. The structure and biological function of Piezo1 have been the focus of intense research and some of the molecular structures of Piezo1 channels have been determined using cryoelectron microscopy. Considerable progress has been made in the study of the structural features, physiological significance, and biophysical properties of Piezo1 protein. Piezo1 has a variety of effects, and it plays an essential and irreplaceable role in the maintenance of human life activities and internal environment homeostasis. In addition, Piezo1 channels are related to a quantity of pathophysiological processes, including red blood cell volume regulation, cell division, and innate immunity. Piezo1 channel mutations are associated with a variety of inherited human diseases, such as autosomal recessive congenital lymphangiodysplasia, hereditary stem cell disorders, and autosomal recessive amyotrophy with perinatal respiratory distress syndrome.

However, research on the structure of Piezo1 faces several challenges. The Piezo1 protein’s large predicted size and inclusion of many transmembrane regions make it difficult to express and purify in crystallography. The changes and stability of Piezo1’s structure in different conformation states are still unclear and require more in-depth study. Based on an extensive literature review, we believe that future directions for Piezo1’s function can be explored in the following ways. First, a comparative study of the structure and mechanical gating mechanisms of Piezo1 and Piezo2. Second, the post-translational modifications of Piezo1 regulate its activation. Thirdly, the treatment of human genetic diseases caused by Piezo1 gene mutations. Finally, the structure of Piezo1 in different conformational states was investigated.

There is still much room for in-depth exploration of the crosstalk between Piezo1 and various pathways. YAP, as a classic marker that is also highly expressed in many malignant tumors, has been found to play a synergistic role with Piezo1 in the progression of many diseases and tumors, but the upstream and downstream relationship and activation mode of the two remain to be determined. In addition, whether the downstream effect of Piezo1 activation is related to Ca^2+^ influx, calcium-independent signal transduction, or both, is a question that needs further investigation.

At present, research on the effect of Piezo1 on tumor progression mainly focuses on tumor proliferation, apoptosis and metastasis, while few studies have been done on angiogenesis and cancer stem cells. In fact, as a mechanical ion channel protein, Piezo1 plays an important role in the response of endothelial cells to blood flow stress and shear stress, which is related to the occurrence and development of cardiovascular diseases such as atherosclerosis. The effect of Piezo1 on angiogenesis may not only cause changes in the content of downstream VEGF, HIF-1, and various pro-angiogenic factors, but also need to be further analyzed in combination with physics and fluid dynamics. Also, new studies on the effect of Piezo1 on the stemness of various stem cells are emerging, but few studies extend it to cancer stem cells.

Therapeutic strategies targeting Piezo1 include both pharmacological intervention and gene therapy. Specific small molecule compounds and drugs have been found to inhibit Piezo1’s activity, thereby inhibiting tumor growth and metastasis. Gene therapy is also being explored to suppress the expression of Piezo1 to stop tumor growth and metastasis. Although targeting Piezo1 to treat tumors is a promising therapeutic approach, the detailed molecular mechanisms that regulate Piezo1 and its regulation of downstream targets are still unclear. The underlying mechanisms still need to be further explored. In addition, developing specific direct Piezo1 antagonists is also an area for further research in the future. At the same time, Piezo1-regulated Ca^2+^ influx extensively affects its downstream pathways due to the signaling cascade, and the development of drugs targeting the major downstream pathways is a possible alternative when other approaches fail. In addition, Piezo1 exhibits different and complex roles in different tumors, indicating that there are other potential pathways that can work together with Piezo1. Also, some studies have used mice to explore what Piezo1 works for, but whether it works the same way in humans remains to be seen. In summary, how to effectively regulate the expression or activity of Piezo1, inhibit tumor progression, and buy enough time for later treatment needs further study.

Taken together, Piezo1 plays an important role in the normal physiological function of the body and the development and evolution of diseases. In addition, Piezo1 is abnormally expressed in a variety of tumors, which is involved in regulating the malignant behavior of tumors and the formation of TIME, and is related to the prognosis of patients. Therefore, Piezo1 is a promising tumor biomarker and therapeutic target.

## References

[B1] Albarrán-Juárez J. Iring A. Wang S. Joseph S. Grimm M. Strilic B. (2018a). Piezo1 and G(q)/G(11) promote endothelial inflammation depending on flow pattern and integrin activation. J. Exp. Med. 215 (10), 2655–2672. 10.1084/jem.20180483 30194266 PMC6170174

[B2] Albarrán-Juárez J. Iring A. Wang S. Joseph S. Grimm M. Strilic B. (2018b). Piezo1 and Gq/G11 promote endothelial inflammation depending on flow pattern and integrin activation. J. Exp. Med. 215 (10), 2655–2672. 10.1084/jem.20180483 30194266 PMC6170174

[B3] Atcha H. Jairaman A. Holt J. R. Meli V. S. Nagalla R. R. Veerasubramanian P. K. (2021). Mechanically activated ion channel Piezo1 modulates macrophage polarization and stiffness sensing. Nat. Commun. 12 (1), 3256. 10.1038/s41467-021-23482-5 34059671 PMC8167181

[B4] Ayata P. Schaefer A. (2020). Innate sensing of mechanical properties of brain tissue by microglia. Curr. Opin. Immunol. 62, 123–130. 10.1016/j.coi.2020.01.003 32058296 PMC7067639

[B5] Aykut B. Chen R. Kim J. I. Wu D. Shadaloey S. A. A. Abengozar R. (2020). Targeting Piezo1 unleashes innate immunity against cancer and infectious disease. Sci. Immunol. 5 (50), eabb5168. 10.1126/sciimmunol.abb5168 32826342

[B6] Bae M. H. Park C. J. Suh C. (2021). Increased monocytic myeloid-derived suppressor cells in whole blood predict poor prognosis in patients with plasma cell myeloma. J. Clin. Med. 10 (20), 4717. 10.3390/jcm10204717 34682840 PMC8540224

[B7] Bagriantsev S. N. Gracheva E. O. Gallagher P. G. (2014). Piezo proteins: regulators of mechanosensation and other cellular processes. J. Biol. Chem. 289 (46), 31673–31681. 10.1074/jbc.R114.612697 25305018 PMC4231648

[B8] Batlle E. Massagué J. (2019). Transforming growth factor-β signaling in immunity and cancer. Immunity 50 (4), 924–940. 10.1016/j.immuni.2019.03.024 30995507 PMC7507121

[B9] Beech D. J. Kalli A. C. (2019a). Force sensing by piezo channels in cardiovascular health and disease. Arterioscler. Thromb. Vasc. Biol. 39 (11), 2228–2239. 10.1161/atvbaha.119.313348 31533470 PMC6818984

[B10] Beech D. J. Kalli A. C. (2019b). Force sensing by piezo channels in cardiovascular health and disease. Arteriosclerosis, thrombosis, Vasc. Biol. 39 (11), 2228–2239. 10.1161/ATVBAHA.119.313348 PMC6818984 31533470

[B11] Botello-Smith W. M. Jiang W. Zhang H. Ozkan A. D. Lin Y. C. Pham C. N. (2019). A mechanism for the activation of the mechanosensitive Piezo1 channel by the small molecule Yoda1. Nat. Commun. 10 (1), 4503. 10.1038/s41467-019-12501-1 31582801 PMC6776524

[B12] Bruno L. Karagil S. Mahmood A. Elbediwy A. Stolinski M. Mackenzie F. E. (2021). Mechanosensing and the Hippo pathway in microglia: a potential link to alzheimer’s disease pathogenesis? Cells 10 (11), 3144. 10.3390/cells10113144 34831369 PMC8622675

[B13] Cai G. Lu Y. Zhong W. Wang T. Li Y. Ruan X. (2023). Piezo1-mediated M2 macrophage mechanotransduction enhances bone formation through secretion and activation of transforming growth factor-β1. Cell Prolif. 56 (9), e13440. 10.1111/cpr.13440 36880296 PMC10472522

[B14] Carragher N. O. Walker S. M. Scott Carragher L. A. Harris F. Sawyer T. K. Brunton V. G. (2006). Calpain 2 and Src dependence distinguishes mesenchymal and amoeboid modes of tumour cell invasion: a link to integrin function. Oncogene 25 (42), 5726–5740. 10.1038/sj.onc.1209582 16652152

[B15] Cerami E. Gao J. Dogrusoz U. Gross B. E. Sumer S. O. Aksoy B. A. (2012). The cBio cancer genomics portal: an open platform for exploring multidimensional cancer genomics data. Cancer Discov. 2 (5), 401–404. 10.1158/2159-8290.CD-12-0095 22588877 PMC3956037

[B16] Chamberlain G. Fox J. Ashton B. Middleton J. (2007). Concise review: mesenchymal stem cells: their phenotype, differentiation capacity, immunological features, and potential for homing. Stem Cells 25 (11), 2739–2749. 10.1634/stemcells.2007-0197 17656645

[B17] Chen L. Yan Y. Kong F. G. Wang J. K. Zeng J. Fang Z. (2022b). Contribution of oxidative stress induced by sonodynamic therapy to the calcium homeostasis imbalance enhances macrophage infiltration in glioma cells. Cancers 14 (8), 2036. 10.3390/cancers14082036 35454942 PMC9027216

[B18] Chen S. Cao R. Xiang L. Li Z. Chen H. Zhang J. (2023). Research progress in nucleus-targeted tumor therapy. Biomater. Sci. 19 (11), 6436–6456. 10.1039/d3bm01116j 37609783

[B19] Chen S. Zhang S. Wang Z. Li J. Yuan Y. Li T. (2022a). Purine metabolism-related gene expression signature predicts survival outcome and indicates immune microenvironment profile of gliomas. Front. Pharmacol. 13, 1038272. 10.3389/fphar.2022.1038272 36438805 PMC9685320

[B20] Chen X. Chen J. (2022). miR-10b-5p-mediated upregulation of PIEZO1 predicts poor prognosis and links to purine metabolism in breast cancer. Genomics 114 (3), 110351. 10.1016/j.ygeno.2022.110351 35351580

[B21] Chen X. Wanggou S. Bodalia A. Zhu M. Dong W. F. Fan J. J. (2018). A feedforward mechanism mediated by mechanosensitive ion channel Piezo1 and tissue mechanics promotes glioma aggression. Neuron 100 (4), 799–815. 10.1016/j.neuron.2018.09.046 30344046

[B22] Chen X.-H. Wang A. Chu A.-N. Gong Y.-H. Yuan Y. (2019a). Mucosa-associated microbiota in gastric cancer tissues compared with non-cancer tissues. Front. Microbiol. 10, 1261. 10.3389/fmicb.2019.01261 31231345 PMC6560205

[B23] Chen Y. Song Y. Du W. Gong L. Chang H. Zou Z. (2019b). Tumor-associated macrophages: an accomplice in solid tumor progression. J. Biomed. Sci. 26 (1), 78. 10.1186/s12929-019-0568-z 31629410 PMC6800990

[B24] Chiba T. Zheng Y. W. Kita K. Yokosuka O. Saisho H. Onoidera M. (2007). Enhanced self-renewal capability in hepatic stem/progenitor cells drives cancer initiation. Gastroenterology 133 (3), 937–950. 10.1053/j.gastro.2007.06.016 17673212

[B25] Choi H. J. Jhe Y. L. Kim J. Lim J. Y. Lee J. E. Shin M. K. (2020). FoxM1-dependent and fatty acid oxidation-mediated ROS modulation is a cell-intrinsic drug resistance mechanism in cancer stem-like cells. Redox Biol. 36, 101589. 10.1016/j.redox.2020.101589 32521504 PMC7286985

[B26] Coste B. Mathur J. Schmidt M. Earley T. J. Ranade S. Petrus M. J. (2010). Piezo1 and Piezo2 are essential components of distinct mechanically activated cation channels. Science 330 (6000), 55–60. 10.1126/science.1193270 20813920 PMC3062430

[B27] Dela Justina V. de Freitas R. A. Arishe O. O. Giachini F. R. Webb R. C. Priviero F. (2023). Piezo1 activation induces relaxation of the pudendal artery and corpus cavernosum. Front. Physiol. 14, 998951. 10.3389/fphys.2023.998951 36846322 PMC9950814

[B28] Del Bello B. Gamberucci A. Marcolongo P. Maellaro E. (2022). The autophagy inducer trehalose stimulates macropinocytosis in NF1-deficient glioblastoma cells. Cancer Cell Int. 22 (1), 232. 10.1186/s12935-022-02652-5 35864494 PMC9306097

[B29] Delire B. Stärkel P. (2015). The Ras/MAPK pathway and hepatocarcinoma: pathogenesis and therapeutic implications. Eur. J. Clin. Investigation 45 (6), 609–623. 10.1111/eci.12441 25832714

[B30] Dienes B. Bazsó T. Szabó L. Csernoch L. (2023). The role of the Piezo1 mechanosensitive channel in the musculoskeletal system. Int. J. Mol. Sci. 24 (7), 6513. 10.3390/ijms24076513 37047487 PMC10095409

[B31] Douguet D. Patel A. Xu A. Vanhoutte P. M. Honoré E. (2019). Piezo ion channels in cardiovascular mechanobiology. Trends Pharmacol. Sci. 40 (12), 956–970. 10.1016/j.tips.2019.10.002 31704174

[B32] Du G. Li L. Zhang X. Liu J. Hao J. Zhu J. (2020). Roles of TRPV4 and piezo channels in stretch-evoked Ca(2+) response in chondrocytes. Exp. Biol. Med. (Maywood) 245 (3), 180–189. 10.1177/1535370219892601 31791130 PMC7045327

[B33] Evans E. L. Cuthbertson K. Endesh N. Rode B. Blythe N. M. Hyman A. J. (2018). Yoda1 analogue (Dooku1) which antagonizes Yoda1-evoked activation of Piezo1 and aortic relaxation. Br. J. Pharmacol. 175 (10), 1744–1759. 10.1111/bph.14188 29498036 PMC5913400

[B34] Evtugina N. G. Peshkova A. D. Khabirova A. I. Andrianova I. A. Abdullayeva S. Ayombil F. (2023). Activation of Piezo1 channels in compressed red blood cells augments platelet-driven contraction of blood clots. J. Thromb. Haemost. 21 (9), 2418–2429. 10.1016/j.jtha.2023.05.022 37268065 PMC10949619

[B35] Fan L. L. Wei A. H. Gao Z. H. Mu X. P. (2023). Current progress of mesenchymal stem cell membrane-camouflaged nanoparticles for targeted therapy. Biomed. Pharmacother. 161, 114451. 10.1016/j.biopha.2023.114451 36870279

[B36] Fang X. Z. Zhou T. Xu J. Q. Wang Y. X. Sun M. M. He Y. J. (2021). Structure, kinetic properties and biological function of mechanosensitive Piezo channels. Cell Biosci. 11 (1), 13. 10.1186/s13578-020-00522-z 33422128 PMC7796548

[B37] Feng X. McDonald J. M. (2011). Disorders of bone remodeling. Annu. Rev. Pathol. 6, 121–145. 10.1146/annurev-pathol-011110-130203 20936937 PMC3571087

[B38] Filser M. Giansily-Blaizot M. Grenier M. Monedero Alonso D. Bouyer G. Pérès L. (2021). Increased incidence of germline PIEZO1 mutations in individuals with idiopathic erythrocytosis. Blood 137 (13), 1828–1832. 10.1182/blood.2020008424 33181827

[B39] Fresno Vara J. A. Casado E. de Castro J. Cejas P. Belda-Iniesta C. González-Barón M. (2004). PI3K/Akt signalling pathway and cancer. Cancer Treat. Rev. 30 (2), 193–204. 10.1016/j.ctrv.2003.07.007 15023437

[B40] Gao L. Ji Y. Wang L. L. He M. J. Yang X. J. Qiu Y. B. (2021). Suppression of esophageal squamous cell carcinoma development by mechanosensitive protein Piezo1 downregulation. Acs Omega 6 (15), 10196–10206. 10.1021/acsomega.1c00505 34056174 PMC8153669

[B41] Gibson J. S. Stewart G. W. (2023). A critical role for altered red cell cation permeability in pathogenesis of sickle cell disease and other haemolytic anaemias. Br. J. Haematol. 202 (3), 462–464. 10.1111/bjh.18832 37096935

[B42] Gilmore T. D. (2003). The Re1/NF-kappa B/I kappa B signal transduction pathway and cancer. Cancer Treat. Res. 115, 241–265. 12613200

[B43] Giron-Ceron D. Jaumouillé V. (2023). The mechanosensor Piezo1 rings the alarm on epithelial intruders. Trends Biochem. Sci. 48 (6), 500–502. 10.1016/j.tibs.2023.03.001 36959017

[B44] Gomes A. M. Soares M. V. D. Ribeiro P. Caldas J. Póvoa V. Martins L. R. (2014). Adult B-cell acute lymphoblastic leukemia cells display decreased PTEN activity and constitutive hyperactivation of PI3K/Akt pathway despite high PTEN protein levels. Haematologica 99 (6), 1062–1068. 10.3324/haematol.2013.096438 24561792 PMC4040910

[B45] Gottlieb P. A. Bae C. Sachs F. (2012). Gating the mechanical channel Piezo1: a comparison between whole-cell and patch recording. Channels (Austin) 6 (4), 282–289. 10.4161/chan.21064 22790451 PMC3508907

[B46] Green D. R. Reed J. C. (1998). Mitochondria and apoptosis. Science 281 (5381), 1309–1312. 10.1126/science.281.5381.1309 9721092

[B47] Greenlee J. D. Liu K. Lopez-Cavestany M. King M. R. (2022). Piezo1 mechano-activation is augmented by resveratrol and differs between colorectal cancer cells of primary and metastatic origin. Molecules 27 (17), 5430. 10.3390/molecules27175430 36080197 PMC9458129

[B48] Griffin M. F. Talbott H. E. Guardino N. J. Guo J. L. Spielman A. F. Chen K. (2023). Piezo inhibition prevents and rescues scarring by targeting the adipocyte to fibroblast transition. bioRxiv 03 (04),535302. 10.1101/2023.04.03.535302

[B49] Gross O. Thomas C. J. Guarda G. Tschopp J. (2011). The inflammasome: an integrated view. Immunol. Rev. 243 (1), 136–151. 10.1111/j.1600-065X.2011.01046.x 21884173

[B50] Guo Y. R. MacKinnon R. (2017). Structure-based membrane dome mechanism for Piezo mechanosensitivity. Elife 6, e33660. 10.7554/eLife.33660 29231809 PMC5788504

[B51] Halder G. Dupont S. Piccolo S. (2012). Transduction of mechanical and cytoskeletal cues by YAP and TAZ. Nat. Rev. Mol. Cell Biol. 13 (9), 591–600. 10.1038/nrm3416 22895435

[B52] Hamza A. Amit J. Elizabeth L. E. Medha M. P. Michael D. C. Wendy F. L. (2021). Ion channel mediated mechanotransduction in immune cells. Curr. Opin. Solid State Mater Sci. 25 (6), 100951. 10.1016/j.cossms.2021.100951 35645593 PMC9131931

[B53] Han Y. Liu C. Zhang D. Men H. Huo L. Geng Q. (2019). Mechanosensitive ion channel Piezo1 promotes prostate cancer development through the activation of the Akt/mTOR pathway and acceleration of cell cycle. Int. J. Oncol. 55 (3), 629–644. 10.3892/ijo.2019.4839 31322184 PMC6685593

[B54] Hanahan D. Weinberg R. A. (2011). Hallmarks of cancer: the next generation. Cell 144 (5), 646–674. 10.1016/j.cell.2011.02.013 21376230

[B55] Hanchard N. A. Wonkam A. (2021). Iron"ing out hemophagocytosis through PIEZO1. Cell 184 (4), 856–858. 10.1016/j.cell.2021.01.038 33606984 PMC9979154

[B56] Hasegawa K. Fujii S. Matsumoto S. Tajiri Y. Kikuchi A. Kiyoshima T. (2021). YAP signaling induces PIEZO1 to promote oral squamous cell carcinoma cell proliferation. J. Pathology 253 (1), 80–93. 10.1002/path.5553 32985688

[B57] Hinshaw D. C. Benavides G. A. Metge B. J. Swain C. A. Kammerud S. C. Alsheikh H. A. (2023). Hedgehog signaling regulates Treg to Th17 conversion through metabolic rewiring in breast cancer. Cancer Immunol. Res. 11 (5), 687–702. 10.1158/2326-6066.Cir-22-0426 37058110 PMC10159910

[B58] Hirata Y. Cai R. Volchuk A. Steinberg B. E. Saito Y. Matsuzawa A. (2023). Lipid peroxidation increases membrane tension, Piezo1 gating, and cation permeability to execute ferroptosis. Curr. Biol. 33 (7), 1282–1294.e5. 10.1016/j.cub.2023.02.060 36898371

[B59] Holohan C. Van Schaeybroeck S. Longley D. B. Johnston P. G. (2013). Cancer drug resistance: an evolving paradigm. Nat. Rev. Cancer 13 (10), 714–726. 10.1038/nrc3599 24060863

[B60] Hope J. M. Lopez-Cavestany M. Wang W. J. Reinhart-King C. A. King M. R. (2019). Activation of Piezo1 sensitizes cells to TRAIL-mediated apoptosis through mitochondrial outer membrane permeability. Cell Death Dis. 10 (11), 837. 10.1038/s41419-019-2063-6 31685811 PMC6828775

[B61] Huang Z. C. Sun Z. Q. Zhang X. Y. Niu K. Wang Y. Zheng J. (2019). Loss of stretch-activated channels, PIEZOs, accelerates non-small cell lung cancer progression and cell migration. Biosci. Rep. 39 (3), BSR20181679. 10.1042/bsr20181679 30745454 PMC6430724

[B62] Huber M. A. Azoitei N. Baumann B. Grünert S. Sommer A. Pehamberger H. (2004). NF-kappaB is essential for epithelial-mesenchymal transition and metastasis in a model of breast cancer progression. J. Clin. Investigation 114 (4), 569–581. 10.1172/JCI21358 PMC503772 15314694

[B63] Isogai T. van der Kammen R. Leyton-Puig D. Kedziora K. M. Jalink K. Innocenti M. (2015). Initiation of lamellipodia and ruffles involves cooperation between mDia1 and the Arp2/3 complex. J. Cell Sci. 128 (20), 3796–3810. 10.1242/jcs.176768 26349808

[B64] Jairaman A. Othy S. Dynes J. L. Yeromin A. V. Zavala A. Greenberg M. L. (2021). Piezo1 channels restrain regulatory T cells but are dispensable for effector CD4(+) T cell responses. Sci. Adv. 7 (28), eabg5859. 10.1126/sciadv.abg5859 34233878 PMC8262815

[B65] Jebari-Benslaiman S. Galicia-García U. Larrea-Sebal A. Olaetxea J. R. Alloza I. Vandenbroeck K. (2022). Pathophysiology of atherosclerosis. Int. J. Mol. Sci. 23 (6), 3346. 10.3390/ijms23063346 35328769 PMC8954705

[B66] Jiang F. Yin K. Wu K. Zhang M. Wang S. Cheng H. (2021b). The mechanosensitive Piezo1 channel mediates heart mechano-chemo transduction. Nat. Commun. 12 (1), 869. 10.1038/s41467-021-21178-4 33558521 PMC7870949

[B67] Jiang L. Zhao Y. D. Chen W. X. (2017). The function of the novel mechanical activated ion channel Piezo1 in the human osteosarcoma cells. Med. Sci. Monit. 23, 5070–5082. 10.12659/msm.906959 29065102 PMC5665612

[B68] Jiang X. Stockwell B. R. Conrad M. (2021c). Ferroptosis: mechanisms, biology and role in disease. Nat. Rev. Mol. Cell Biol. 22 (4), 266–282. 10.1038/s41580-020-00324-8 33495651 PMC8142022

[B69] Jiang Y. Wang C. X. Wang Y. L. Zhang W. Liu L. K. Cheng J. (2023). Prognostic role of CD11b(+) myeloid-derived suppressor cells in oral squamous cell carcinoma. Archives Med. Sci. 19 (1), 171–179. 10.5114/aoms/116683 PMC9897095 36817676

[B70] Jiang Y. Yang X. Jiang J. Xiao B. (2021a). Structural designs and mechanogating mechanisms of the mechanosensitive piezo channels. Trends Biochem. Sci. 46 (6), 472–488. 10.1016/j.tibs.2021.01.008 33610426

[B71] Karamatic C. V. Tilley L. A. Satchwell T. J. AlSubhi S. A. Jones B. Spring F. A. (2023). Missense mutations in PIEZO1, which encodes the Piezo1 mechanosensor protein, define Er red blood cell antigens. Blood 141 (2), 135–146. 10.1182/blood.2022016504 36122374 PMC10644042

[B72] Kauffmann-Zeh A. Rodriguez-Viciana P. Ulrich E. Gilbert C. Coffer P. Downward J. (1997). Suppression of c-Myc-induced apoptosis by Ras signalling through PI(3)K and PKB. Nature 385 (6616), 544–548. 10.1038/385544a0 9020362

[B73] Kim H. S. Suh J. S. Jang Y. K. Ahn S. H. Choi G. H. Yang J. Y. (2022b). Forster resonance energy transfer-based single-cell imaging reveals piezo1-induced Ca2+ flux mediates membrane ruffling and cell survival. Front. Cell Dev. Biol. 10, 865056. 10.3389/fcell.2022.865056 35646889 PMC9136143

[B74] Kim O.-H. Choi Y. W. Hong S. A. Hong M. Chang I. H. Lee H. J. (2022a). Fluid shear stress facilitates prostate cancer metastasis through Piezo1-Src-YAP axis. Life Sci. 308, 120936. 10.1016/j.lfs.2022.120936 36084759

[B75] Kim S. E. Coste B. Chadha A. Cook B. Patapoutian A. (2012). The role of Drosophila Piezo in mechanical nociception. Nature 483 (7388), 209–212. 10.1038/nature10801 22343891 PMC3297676

[B76] Knoblauch S. V. Desai S. H. Dombroski J. A. Sarna N. S. Hope J. M. King M. R. (2023). Chemical activation and mechanical sensitization of Piezo1 enhance TRAIL-mediated apoptosis in glioblastoma cells. ACS Omega 8 (19), 16975–16986. 10.1021/acsomega.3c00705 37214705 PMC10193566

[B77] Köster P. DeFalco T. A. Zipfel C. (2022). Ca(2+) signals in plant immunity. Embo J. 41 (12), e110741. 10.15252/embj.2022110741 35560235 PMC9194748

[B78] Kubiak A. Zieliński T. Pabijan J. Lekka M. (2020). Nanomechanics in monitoring the effectiveness of drugs targeting the cancer cell cytoskeleton. Int. J. Mol. Sci. 21 (22), 8786. 10.3390/ijms21228786 33233645 PMC7699791

[B79] Kuntze A. Goetsch O. Fels B. Najder K. Unger A. Wilhelmi M. (2020). Protonation of Piezo1 impairs cell-matrix interactions of pancreatic stellate cells. Front. Physiology 11, 89. 10.3389/fphys.2020.00089 PMC7033545 32116794

[B80] Kuriyama M. Hirose H. Masuda T. Shudou M. Arafiles J. V. Imanishi M. (2022). Piezo1 activation using Yoda1 inhibits macropinocytosis in A431 human epidermoid carcinoma cells. Sci. Rep. 12 (1), 6322. 10.1038/s41598-022-10153-8 35428847 PMC9012786

[B81] Kwak K. Sohn H. George R. Torgbor C. Manzella-Lapeira J. Brzostowski J. (2023). B cell responses to membrane-presented antigens require the function of the mechanosensitive cation channel Piezo1. Sci. Signal 16 (804), eabq5096. 10.1126/scisignal.abq5096 37751477 PMC10691204

[B82] Leng S. Zhang X. Wang S. Qin J. Liu Q. Liu A. (2022). Ion channel Piezo1 activation promotes aerobic glycolysis in macrophages. Front. Immunol. 13, 976482. 10.3389/fimmu.2022.976482 36119083 PMC9479104

[B83] Li C. Y. Rezania S. Kammerer S. Sokolowski A. Devaney T. Gorischek A. (2015). Piezo1 forms mechanosensitive ion channels in the human MCF-7 breast cancer cell line. Sci. Rep. 5, 8364. 10.1038/srep08364 25666479 PMC4322926

[B84] Li J. Zhang Y. Lou Z. Li M. Cui L. Yang Z. (2022a). Magnetic nanobubble mechanical stress induces the Piezo1-Ca2+ -BMP2/Smad pathway to modulate neural stem cell fate and MRI/ultrasound dual imaging surveillance for ischemic stroke. Small Weinheim Der Bergstrasse, Ger. 18 (23), e2201123. 10.1002/smll.202201123 35555970

[B85] Li M. Zhang X. Wang M. M. Wang Y. H. Qian J. L. Xing X. X. (2022c). Activation of Piezo1 contributes to matrix stiffness-induced angiogenesis in hepatocellular carcinoma. Cancer Commun. 42 (11), 1162–1184. 10.1002/cac2.12364 PMC9648387 36181398

[B86] Li R. Wang D. Li H. Lei X. Liao W. Liu X.-Y. (2023a). Identification of Piezo1 as a potential target for therapy of colon cancer stem-like cells. Discov. Oncol. 14 (1), 95. 10.1007/s12672-023-00712-4 37306789 PMC10260724

[B87] Li S. Yu J. Huber A. Kryczek I. Wang Z. Jiang L. (2022b). Metabolism drives macrophage heterogeneity in the tumor microenvironment. Cell Rep. 39 (1), 110609. 10.1016/j.celrep.2022.110609 35385733 PMC9052943

[B88] Li Y. M. Xu C. Sun B. Zhong F. J. Cao M. Yang L. Y. (2022e). Piezo1 promoted hepatocellular carcinoma progression and EMT through activating TGF-β signaling by recruiting Rab5c. Cancer Cell Int. 22 (1), 162. 10.1186/s12935-022-02574-2 35461277 PMC9035260

[B89] Li Y. M. Xu C. Sun B. Zhong F. J. Cao M. M. Yang L. Y. (2022d). Piezo1 promoted hepatocellular carcinoma progression and EMT through activating TGF-beta signaling by recruiting Rab5c. Cancer Cell Int. 22 (1), 19. 10.1186/s12935-022-02574-2 35461277 PMC9035260

[B90] Li Z. H. Xi J. C. Li B. K. Liu Y. Q. Wang G. Y. Yu B. (2023b). SHP-2-induced M2 polarization of tumor associated macrophages via IL-4 regulate colorectal cancer progression. Front. Oncol. 13, 1027575. 10.3389/fonc.2023.1027575 36776333 PMC9909964

[B91] Lin C. Zheng X. Lin S. Zhang Y. Wu J. Li Y. (2022a). Mechanotransduction regulates the interplays between alveolar epithelial and vascular endothelial cells in lung. Front. Physiol. 13, 818394. 10.3389/fphys.2022.818394 35250619 PMC8895143

[B92] Lin C. Y. Song X. Ke Y. Raha A. Wu Y. N. Wasi M. (2022b). Yoda1 enhanced low-magnitude high-frequency vibration on osteocytes in regulation of MDA-MB-231 breast cancer cell migration. Cancers 14 (14), 3395. 10.3390/cancers14143395 35884459 PMC9324638

[B93] Liou G.-Y. Storz P. (2010). Reactive oxygen species in cancer. Free Radic. Res. 44 (5), 479–496. 10.3109/10715761003667554 20370557 PMC3880197

[B94] Liu C.-J. Ma Z.-Z. Gong W.-Z. Mao X.-H. Wen H.-Q. Wang X.-H. (2023). The role of purine metabolism-related genes PPAT and IMPDH1 in the carcinogenesis of intrahepatic cholangiocarcinoma based on metabonomic and bioinformatic analyses. J. Oncol. 2023, 5141836. 10.1155/2023/5141836 36711025 PMC9883099

[B95] Liu D. D. Han C. C. Wan H. F. He F. Xu H. Y. Wei S. H. (2016). Effects of inhibiting PI3K-Akt-mTOR pathway on lipid metabolism homeostasis in goose primary hepatocytes. Animal Int. J. Animal Biosci. 10 (8), 1319–1327. 10.1017/S1751731116000380 26956906

[B96] Liu S. Xu X. Fang Z. Ning Y. Deng B. Pan X. (2021a). Piezo1 impairs hepatocellular tumor growth via deregulation of the MAPK-mediated YAP signaling pathway. Cell Calcium 95, 102367. 10.1016/j.ceca.2021.102367 33610907

[B97] Liu X. J. Wu Y. Li M. T. Hao J. Y. Wang Q. Zeng X. F. (2021b). Plasticity of Treg and imbalance of Treg/Th17 cells in patients with systemic sclerosis modified by FK506. Int. J. Immunopathol. Pharmacol. 35, 2058738421998086. 10.1177/2058738421998086 33631989 PMC7917869

[B98] Liu Y. L. Tian H. T. Hu Y. X. Cao Y. L. Song H. Lan S. H. (2022). Mechanosensitive Piezo1 is crucial for periosteal stem cell-mediated fracture healing. Int. J. Biol. Sci. 18 (10), 3961–3980. 10.7150/ijbs.71390 35844802 PMC9274506

[B99] Lohia R. Allegrini B. Berry L. Guizouarn H. Cerdan R. Abkarian M. (2023). Pharmacological activation of PIEZO1 in human red blood cells prevents Plasmodium falciparum invasion. Cell Mol. Life Sci. 80 (5), 124. 10.1007/s00018-023-04773-0 37071200 PMC10113305

[B100] Lopez-Cavestany M. Hahn S. B. Hope J. M. Reckhorn N. T. Greenlee J. D. Schwager S. C. (2023). Matrix stiffness induces epithelial-to-mesenchymal transition via Piezo1-regulated calcium flux in prostate cancer cells. Iscience 26 (4), 106275. 10.1016/j.isci.2023.106275 36950111 PMC10025097

[B101] Lukacs V. Mathur J. Mao R. Bayrak-Toydemir P. Procter M. Cahalan S. M. (2015). Impaired PIEZO1 function in patients with a novel autosomal recessive congenital lymphatic dysplasia. Nat. Commun. 6, 8329. 10.1038/ncomms9329 26387913 PMC4578306

[B102] Lv B. Wang Y. Ma D. Cheng W. Liu J. Yong T. (2022). Immunotherapy: reshape the tumor immune microenvironment. Front. Immunol. 13, 844142. 10.3389/fimmu.2022.844142 35874717 PMC9299092

[B103] Lv J. W. Li J. Y. Luo L. N. Wang Z. X. Chen Y. P. (2019). Comparative safety and efficacy of anti-PD-1 monotherapy, chemotherapy alone, and their combination therapy in advanced nasopharyngeal carcinoma: findings from recent advances in landmark trials. J. Immunother. Cancer 7 (1), 159. 10.1186/s40425-019-0636-7 31238988 PMC6593483

[B104] Malko P. Jia X. Wood I. Jiang L.-H. (2023). Piezo1 channel-mediated Ca2+ signaling inhibits lipopolysaccharide-induced activation of the NF-κB inflammatory signaling pathway and generation of TNF-α and IL-6 in microglial cells. Glia 71 (4), 848–865. 10.1002/glia.24311 36447422

[B105] Mao Z. Y. Zhang J. H. Shi Y. H. Li W. Shi H. Ji R. B. (2020). CXCL5 promotes gastric cancer metastasis by inducing epithelial-mesenchymal transition and activating neutrophils. Oncogenesis 9 (7), 63. 10.1038/s41389-020-00249-z 32632106 PMC7338464

[B106] McHugh B. J. Murdoch A. Haslett C. Sethi T. (2012). Loss of the integrin-activating transmembrane protein Fam38A (Piezo1) promotes a switch to a reduced integrin-dependent mode of cell migration. Plos One 7 (7), e40346. 10.1371/journal.pone.0040346 22792288 PMC3390408

[B107] Mills C. D. Kincaid K. Alt J. M. Heilman M. J. Hill A. M. (2000). M-1/M-2 macrophages and the Th1/Th2 paradigm. J. Immunol. 164 (12), 6166–6173. 10.4049/jimmunol.164.12.6166 10843666

[B108] Mousawi F. Peng H. S. Li J. Sreenivasan P. Roger S. Zhao H. C. (2020). Chemical activation of the Piezo1 channel drives mesenchymal stem cell migration via inducing ATP release and activation of P2 receptor purinergic signaling. Stem Cells 38 (3), 410–421. 10.1002/stem.3114 31746084 PMC7064961

[B109] Mukhopadhyay A. Tsukasaki Y. Chan W. C. Le J. P. Kwok M. L. Zhou J. (2024). trans-Endothelial neutrophil migration activates bactericidal function via Piezo1 mechanosensing. Immunity 57 (1), 52–67.e10. 10.1016/j.immuni.2023.11.007 38091995 PMC10872880

[B110] Mulhall E. M. Gharpure A. Lee R. M. Dubin A. E. Aaron J. S. Marshall K. L. (2023). Direct observation of the conformational states of PIEZO1. Nature 620 (7976), 1117–1125. 10.1038/s41586-023-06427-4 37587339 PMC10468401

[B111] Mun E. J. Babiker H. M. Weinberg U. Kirson E. D. Von Hoff D. D. (2018). Tumor-treating fields: a fourth modality in cancer treatment. Clin. Cancer Res. 24 (2), 266–275. 10.1158/1078-0432.Ccr-17-1117 28765323

[B112] Nader E. Conran N. Leonardo F. C. Hatem A. Boisson C. Carin R. (2023). Piezo1 activation augments sickling propensity and the adhesive properties of sickle red blood cells in a calcium-dependent manner. Br. J. Haematol. 202 (3), 657–668. 10.1111/bjh.18799 37011913

[B113] Nageswara Rao A. A. Scafidi J. Wells E. M. Packer R. J. (2012). Biologically targeted therapeutics in pediatric brain tumors. Pediatr. Neurol. 46 (4), 203–211. 10.1016/j.pediatrneurol.2012.02.005 22490764 PMC3654250

[B114] Nakamichi R. Ma S. Nonoyama T. Chiba T. Kurimoto R. Ohzono H. (2022). The mechanosensitive ion channel PIEZO1 is expressed in tendons and regulates physical performance. Sci. Transl. Med. 14 (647), eabj5557. 10.1126/scitranslmed.abj5557 35648809

[B115] Navone S. E. Marfia G. Giannoni A. Beretta M. Guarnaccia L. Gualtierotti R. (2017). Inflammatory mediators and signalling pathways controlling intervertebral disc degeneration. Histology Histopathol. 32 (6), 523–542. 10.14670/HH-11-846 27848245

[B116] Nguyen Ho-Bouldoires T. H. Sollier K. Zamfirov L. Broders-Bondon F. Mitrossilis D. Bermeo S. (2022). Ret kinase-mediated mechanical induction of colon stem cells by tumor growth pressure stimulates cancer progression *in vivo* . Commun. Biol. 5 (1), 137. 10.1038/s42003-022-03079-4 35177769 PMC8854631

[B117] Nishimoto S. Nishida E. (2006). MAPK signalling: ERK5 versus ERK1/2. Embo Rep. 7 (8), 782–786. 10.1038/sj.embor.7400755 16880823 PMC1525153

[B118] O’Callaghan P. Engberg A. Eriksson O. Fatsis-Kavalopoulos N. Stelzl C. Sanchez G. (2022). Piezo1 activation attenuates thrombin-induced blebbing in breast cancer cells. J. Cell Sci. 135 (7), jcs258809. 10.1242/jcs.258809 35274124 PMC9016622

[B119] Oeckinghaus A. Hayden M. S. Ghosh S. (2011). Crosstalk in NF-κB signaling pathways. Nat. Immunol. 12 (8), 695–708. 10.1038/ni.2065 21772278

[B120] Pan X. Wan R. Wang Y. Liu S. He Y. Deng B. (2022). Inhibition of chemically and mechanically activated Piezo1 channels as a mechanism for ameliorating atherosclerosis with salvianolic acid B. Br. J. Pharmacol. 179 (14), 3778–3814. 10.1111/bph.15826 35194776

[B121] Pan Y. Yu Y. Wang X. Zhang T. (2020). Tumor-associated macrophages in tumor immunity. Front. Immunol. 11, 583084. 10.3389/fimmu.2020.583084 33365025 PMC7751482

[B122] Panciera T. Azzolin L. Cordenonsi M. Piccolo S. (2017). Mechanobiology of YAP and TAZ in physiology and disease. Nat. Rev. Mol. Cell Biol. 18 (12), 758–770. 10.1038/nrm.2017.87 28951564 PMC6192510

[B123] Park S. M. Park S. H. Ryu K. J. Kim I. K. Han H. Kim H. J. (2019). Downregulation of CHIP promotes ovarian cancer metastasis by inducing Snail-mediated epithelial-mesenchymal transition. Mol. Oncol. 13 (5), 1280–1295. 10.1002/1878-0261.12485 30927556 PMC6487736

[B124] Pavlova Natalya N. Thompson C. B. (2016). The emerging hallmarks of cancer metabolism. Cell Metab. 23 (1), 27–47. 10.1016/j.cmet.2015.12.006 26771115 PMC4715268

[B125] Peyronnet R. Martins J. R. Duprat F. Demolombe S. Arhatte M. Jodar M. (2013). Piezo1-dependent stretch-activated channels are inhibited by Polycystin-2 in renal tubular epithelial cells. EMBO Rep. 14 (12), 1143–1148. 10.1038/embor.2013.170 24157948 PMC3981085

[B126] Pinto V. M. Russo R. Quintino S. Rosato B. E. Marra R. Del Giudice F. (2023). Coinheritance of PIEZO1 variants and multi-locus red blood cell defects account for the symptomatic phenotype in beta-thalassemia carriers. Am. J. Hematol. 98 (6), E130–E133. 10.1002/ajh.26901 36882369

[B127] Qin L. He T. Chen S. Yang D. Yi W. Cao H. (2021). Roles of mechanosensitive channel Piezo1/2 proteins in skeleton and other tissues. Bone Res. 9 (1), 44. 10.1038/s41413-021-00168-8 34667178 PMC8526690

[B128] Qin X. Ni Z. Jiang J. Liu X. Dong X. Li M. (2022). High-throughput membrane-anchored proteome screening reveals PIEZO1 as a promising antibody-drug target for human esophageal squamous cell carcinoma. Cancer Med. 11 (19), 3700–3713. 10.1002/cam4.4744 35608274 PMC9554447

[B129] Qu S. Q. Li S. T. Hu Z. C. (2020). Upregulation of Piezo1 is a novel prognostic indicator in glioma patients. Cancer Manag. Res. 12, 3527–3536. 10.2147/cmar.S251776 32547190 PMC7244349

[B130] Ranade S. S. Qiu Z. Woo S. H. Hur S. S. Murthy S. E. Cahalan S. M. (2014). Piezo1, a mechanically activated ion channel, is required for vascular development in mice. Proc. Natl. Acad. Sci. U. S. A. 111 (28), 10347–10352. 10.1073/pnas.1409233111 24958852 PMC4104881

[B131] Revathidevi S. Munirajan A. K. (2019). Akt in cancer: mediator and more. Seminars Cancer Biol. 59, 80–91. 10.1016/j.semcancer.2019.06.002 31173856

[B132] Risbud M. V. Shapiro I. M. (2014). Role of cytokines in intervertebral disc degeneration: pain and disc content. Nat. Rev. Rheumatol. 10 (1), 44–56. 10.1038/nrrheum.2013.160 24166242 PMC4151534

[B133] Rode B. Shi J. Endesh N. Drinkhill M. J. Webster P. J. Lotteau S. J. (2017). Piezo1 channels sense whole body physical activity to reset cardiovascular homeostasis and enhance performance. Nat. Commun. 8 (1), 350. 10.1038/s41467-017-00429-3 28839146 PMC5571199

[B134] Rolland L. Torrente A. G. Bourinet E. Maskini D. Drouard A. Chevalier P. (2023). Prolonged Piezo1 activation induces cardiac arrhythmia. Int. J. Mol. Sci. 24 (7), 6720. 10.3390/ijms24076720 37047693 PMC10094979

[B135] Romac J. M. Shahid R. A. Swain S. M. Vigna S. R. Liddle R. A. (2018). Piezo1 is a mechanically activated ion channel and mediates pressure induced pancreatitis. Nat. Commun. 9 (1), 1715. 10.1038/s41467-018-04194-9 29712913 PMC5928090

[B136] Roma-Rodrigues C. Mendes R. Baptista P. V. Fernandes A. R. (2019). Targeting tumor microenvironment for cancer therapy. Int. J. Mol. Sci. 20 (4), 840. 10.3390/ijms20040840 30781344 PMC6413095

[B137] Romero L. O. Massey A. E. Mata-Daboin A. D. Sierra-Valdez F. J. Chauhan S. C. Cordero-Morales J. F. (2019). Dietary fatty acids fine-tune Piezo1 mechanical response. Nat. Commun. 10 (1), 1200. 10.1038/s41467-019-09055-7 30867417 PMC6416271

[B138] Ruiz-Lafuente N. Minguela A. Muro M. Parrado A. (2021). The roles of Cdc42 and Rac1 in the formation of plasma membrane protrusions in cancer epithelial HeLa cells. Mol. Biol. Rep. 48 (5), 4285–4294. 10.1007/s11033-021-06443-5 34110575

[B139] Saotome K. Murthy S. E. Kefauver J. M. Whitwam T. Patapoutian A. Ward A. B. (2018). Structure of the mechanically activated ion channel Piezo1. Nature 554 (7693), 481–486. 10.1038/nature25453 29261642 PMC6010196

[B140] Semenza G. L. (2002). HIF-1 and tumor progression: pathophysiology and therapeutics. Trends Mol. Med. 8 (4), S62–S67. 10.1016/s1471-4914(02)02317-1 11927290

[B141] Shen Q. Hill T. Cai X. Bui L. Barakat R. Hills E. (2021). Physical confinement during cancer cell migration triggers therapeutic resistance and cancer stem cell-like behavior. Cancer Lett. 506, 142–151. 10.1016/j.canlet.2021.01.020 33639204 PMC8112468

[B142] Shi J. Hyman A. J. De Vecchis D. Chong J. Lichtenstein L. Futers T. S. (2020). Sphingomyelinase disables inactivation in endogenous PIEZO1 channels. Cell Rep. 33 (1), 108225. 10.1016/j.celrep.2020.108225 33027663 PMC7539531

[B143] Singh A. Tijore A. Margadant F. Simpson C. Chitkara D. Low B. C. (2021). Enhanced tumor cell killing by ultrasound after microtubule depolymerization. Bioeng. Transl. Med. 6 (3), e10233. 10.1002/btm2.10233 34589605 PMC8459596

[B144] Singh H. (2023). Role of molecular targeted therapeutic drugs in treatment of glioblastoma: a review article. Glob. Med. Genet. 10 (2), 42–47. 10.1055/s-0043-57028 37077370 PMC10110362

[B145] Song S. Zhang Y. Ding T. Ji N. Zhao H. (2020). The dual role of macropinocytosis in cancers: promoting growth and inducing methuosis to participate in anticancer therapies as targets. Front. Oncol. 10, 570108. 10.3389/fonc.2020.570108 33542897 PMC7851083

[B146] Song Y. Chen J. F. Zhang C. Xin L. Li Q. Y. Liu Y. J. (2022). Mechanosensitive channel Piezo1 induces cell apoptosis in pancreatic cancer by ultrasound with microbubbles. Iscience 25 (2), 103733. 10.1016/j.isci.2022.103733 35118354 PMC8792083

[B147] Spaeth E. L. Dembinski J. L. Sasser A. K. Watson K. Klopp A. Hall B. (2009). Mesenchymal stem cell transition to tumor-associated fibroblasts contributes to fibrovascular network expansion and tumor progression. Plos One 4 (4), e4992. 10.1371/journal.pone.0004992 19352430 PMC2661372

[B148] Spokoini R. Kfir-Erenfeld S. Yefenof E. Sionov R. V. (2010). Glycogen synthase kinase-3 plays a central role in mediating glucocorticoid-induced apoptosis. Mol. Endocrinol. Baltim. Md. 24 (6), 1136–1150. 10.1210/me.2009-0466 PMC5417474 20371704

[B149] Stewart T. A. Davis F. M. (2019). Formation and function of mammalian epithelia: roles for mechanosensitive PIEZO1 ion channels. Front. Cell Dev. Biol. 7, 260. 10.3389/fcell.2019.00260 31750303 PMC6843007

[B150] Sun Y. Leng P. Song M. Li D. Guo P. Xu X. (2020a). Piezo1 activates the NLRP3 inflammasome in nucleus pulposus cell-mediated by Ca2+/NF-κB pathway. Int. Immunopharmacol. 85, 106681. 10.1016/j.intimp.2020.106681 32526681

[B151] Sun Y. H. Li M. Liu G. J. Zhang X. Zhi L. H. Zhao J. (2020b). The function of Piezo1 in colon cancer metastasis and its potential regulatory mechanism. J. Cancer Res. Clin. Oncol. 146 (5), 1139–1152. 10.1007/s00432-020-03179-w 32152662 PMC7142063

[B152] Suzuki T. Muraki Y. Hatano N. Suzuki H. Muraki K. (2018). PIEZO1 channel is a potential regulator of synovial sarcoma cell-viability. Int. J. Mol. Sci. 19 (5), 1452. 10.3390/ijms19051452 29757938 PMC5983681

[B153] Syeda R. (2021). Physiology and pathophysiology of mechanically activated PIEZO channels. Annu. Rev. Neurosci. 44, 383–402. 10.1146/annurev-neuro-093020-120939 34236889

[B154] Tadala L. Langenbach D. Dannborg M. Cervantes-Rivera R. Sharma A. Vieth K. (2022). Infection-induced membrane ruffling initiates danger and immune signaling via the mechanosensor PIEZO1. Cell Rep. 40 (6), 111173. 10.1016/j.celrep.2022.111173 35947957

[B155] Talmadge J. E. Fidler I. J. (2010). AACR centennial series: the biology of cancer metastasis: historical perspective. Cancer Res. 70 (14), 5649–5669. 10.1158/0008-5472.Can-10-1040 20610625 PMC4037932

[B156] Tang Y. Zhao C. Zhuang Y. Zhong A. Wang M. Zhang W. (2023). Mechanosensitive Piezo1 protein as a novel regulator in macrophages and macrophage-mediated inflammatory diseases. Front. Immunol. 14, 1149336. 10.3389/fimmu.2023.1149336 37334369 PMC10275567

[B157] Tian B. R. Lin W. F. Zhang Y. (2021). Effects of biomechanical forces on the biological behavior of cancer stem cells. J. Cancer 12 (19), 5895–5902. 10.7150/jca.60893 34476003 PMC8408108

[B158] Tijore A. Yang B. Sheetz M. (2022). Cancer cells can be killed mechanically or with combinations of cytoskeletal inhibitors. Front. Pharmacol. 13, 955595. 10.3389/fphar.2022.955595 36299893 PMC9589226

[B159] Tuomela K. Levings M. K. (2023). Acidity promotes the differentiation of immunosuppressive regulatory T cells. Eur. J. Immunol. 53 (6), e2350511. 10.1002/eji.202350511 37097063

[B160] van Dalen F. J. van Stevendaal M. Fennemann F. L. Verdoes M. Ilina O. (2018). Molecular repolarisation of tumour-associated macrophages. Molecules 24 (1), 9. 10.3390/molecules24010009 30577495 PMC6337345

[B161] Vasileva V. Chubinskiy-Nadezhdin V. (2023). Regulation of PIEZO1 channels by lipids and the structural components of extracellular matrix/cell cytoskeleton. J. Cell Physiol. 238 (5), 918–930. 10.1002/jcp.31001 36947588

[B162] Völkner M. Wagner F. Steinheuer L. M. Carido M. Kurth T. Yazbeck A. (2022). HBEGF-TNF induce a complex outer retinal pathology with photoreceptor cell extrusion in human organoids. Nat. Commun. 13 (1), 6183. 10.1038/s41467-022-33848-y 36261438 PMC9581928

[B163] Wang C. H. Zhang S. S. Liu J. Tian Y. Ma B. Y. Xu S. R. (2020c). Secreted pyruvate kinase M2 promotes lung cancer metastasis through activating the integrin Beta1/FAK signaling pathway. Cell Rep. 30 (6), 1780–1797. 10.1016/j.celrep.2020.01.037 32049010

[B164] Wang L. You X. Lotinun S. Zhang L. Wu N. Zou W. (2020a). Mechanical sensing protein PIEZO1 regulates bone homeostasis via osteoblast-osteoclast crosstalk. Nat. Commun. 11 (1), 282. 10.1038/s41467-019-14146-6 31941964 PMC6962448

[B165] Wang Q. Peng X. Chen Y. Tang X. Qin Y. He M. (2023b). Piezo1 alleviates acetaminophen-induced acute liver injury by activating Nrf2 and reducing mitochondrial reactive oxygen species. Biochem. Biophys. Res. Commun. 652, 88–94. 10.1016/j.bbrc.2023.02.043 36841099

[B166] Wang S. Cao S. Arhatte M. Li D. Shi Y. Kurz S. (2020b). Adipocyte Piezo1 mediates obesogenic adipogenesis through the FGF1/FGFR1 signaling pathway in mice. Nat. Commun. 11 (1), 2303. 10.1038/s41467-020-16026-w 32385276 PMC7211025

[B167] Wang X. Li L. Sun B. Hou X. Song S. Shi C. (2023a). Piezo1-ERK1/2-YAP signaling cascade regulates the proliferation of urine-derived stem cells on collagen gels. Curr. Stem Cell Res. Ther. 19, 103–115. 10.2174/1574888x18666230331123540 36999714

[B168] Wang X. F. Cheng G. Miao Y. Qiu F. Y. Bai L. G. Gao Z. F. (2021). Piezo type mechanosensitive ion channel component 1 facilitates gastric cancer omentum metastasis. J. Cell. Mol. Med. 25 (4), 2238–2253. 10.1111/jcmm.16217 33439514 PMC7882944

[B169] Wang Y. X. Yang H. Jia A. N. Wang Y. F. Yang Q. L. Dong Y. J. (2022). Dendritic cell Piezo1 directs the differentiation of T(H)1 and T-reg cells in cancer. Elife 11, e79957. 10.7554/eLife.79957 35993548 PMC9451538

[B170] Xia H. Huang Y. Zhang L. Luo L. Wang X. Lu Q. (2023). Inhibition of macropinocytosis enhances the sensitivity of osteosarcoma cells to benzethonium chloride. Cancers 15 (3), 961. 10.3390/cancers15030961 36765917 PMC9913482

[B171] Xie T.-X. Xia Z. Zhang N. Gong W. Huang S. (2010). Constitutive NF-kappaB activity regulates the expression of VEGF and IL-8 and tumor angiogenesis of human glioblastoma. Oncol. Rep. 23 (3), 725–732. 10.3892/or_00000690 20127012

[B172] Xing Y. Yang B. He Y. Xie B. Zhao T. Chen J. (2022). Effects of mechanosensitive ion channel Piezo1 on proliferation and osteogenic differentiation of human dental follicle cells. Ann. Anat. 239, 151847. 10.1016/j.aanat.2021.151847 34687906

[B173] Xiong Y. J. Dong L. R. Bai Y. Tang H. Li S. Luo D. (2022). Piezo1 activation facilitates ovarian cancer metastasis via Hippo/YAP signaling axis. Channels 16 (1), 159–166. 10.1080/19336950.2022.2099381 35942515 PMC9367648

[B174] Xu X. Y. Liu Y. H. Hu H. Y. Wang J. S. Cai Y. X. Xie J. (2023). Relationship between cancer stem cell-related SNPs and survival outcomes in patients with primary lung cancer. World J. Surg. Oncol. 21 (1), 243. 10.1186/s12957-023-03064-z 37563730 PMC10416443

[B175] Xu X. Z. (2016). Demystifying mechanosensitive piezo ion channels. Neurosci. Bull. 32 (3), 307–309. 10.1007/s12264-016-0033-x 27164907 PMC5563776

[B176] Yang J. Yuan K. Zhang T. Zhou S. Li W. Chen Z. (2023). Accelerated bone reconstruction by the Yoda1 bilayer membrane via promotion of osteointegration and angiogenesis. Adv. Healthc. Mater 12 (18), e2203105. 10.1002/adhm.202203105 36912184

[B177] Yang X. N. Lu Y. P. Liu J. J. Huang J. K. Liu Y. P. Xiao C. X. (2014). Piezo1 is as a novel trefoil factor family 1 binding protein that promotes gastric cancer cell mobility *in vitro* . Dig. Dis. Sci. 59 (7), 1428–1435. 10.1007/s10620-014-3044-3 24798994

[B178] Yarishkin O. Phuong T. T. T. Baumann J. M. De Ieso M. L. Vazquez-Chona F. Rudzitis C. N. (2021). Piezo1 channels mediate trabecular meshwork mechanotransduction and promote aqueous fluid outflow. J. Physiol. 599 (2), 571–592. 10.1113/jp281011 33226641 PMC7849624

[B179] Youn J.-Y. Wang T. Cai H. (2009). An ezrin/calpain/PI3K/AMPK/eNOS s1179 signaling cascade mediating VEGF-dependent endothelial nitric oxide production. Circulation Res. 104 (1), 50–59. 10.1161/circresaha.108.178467 19038867 PMC2720268

[B180] Zhang C. Yu Y. Huang Q. Tang K. (2019). SIRT6 regulates the proliferation and apoptosis of hepatocellular carcinoma via the ERK1/2 signaling pathway. Mol. Med. Rep. 20 (2), 1575–1582. 10.3892/mmr.2019.10398 31257493 PMC6625461

[B181] Zhang J. L. Zhou Y. H. Huang T. T. Wu F. Liu L. P. Kwan J. S. H. (2018). PIEZO1 functions as a potential oncogene by promoting cell proliferation and migration in gastric carcinogenesis. Mol. Carcinog. 57 (9), 1144–1155. 10.1002/mc.22831 29683214

[B182] Zhang S. Cao S. Gong M. Zhang W. Zhang W. Zhu Z. (2022b). Mechanically activated ion channel Piezo1 contributes to melanoma malignant progression through AKT/mTOR signaling. Cancer Biol. Ther. 23 (1), 336–347. 10.1080/15384047.2022.2060015 36112948 PMC9037449

[B183] Zhang X. N. Gao Y. Zhang X. Y. Guo N. J. Hou W. Q. Wang S. W. (2023a). Detailed curriculum vitae of HER2-targeted therapy. Pharmacol. Ther. 245, 108417. 10.1016/j.pharmthera.2023.108417 37075933

[B184] Zhang X. X. Sun Y. Ma Y. S. Gao C. W. Zhang Y. Z. Yang X. K. (2023b). Tumor-associated M2 macrophages in the immune microenvironment influence the progression of renal clear cell carcinoma by regulating M2 macrophage-associated genes. Front. Oncol. 13, 1157861. 10.3389/fonc.2023.1157861 37361571 PMC10285481

[B185] Zhang Y. Recouvreux M. V. Jung M. Galenkamp K. M. O. Li Y. Zagnitko O. (2021). Macropinocytosis in cancer-associated fibroblasts is dependent on CaMKK2/ARHGEF2 signaling and functions to support tumor and stromal cell fitness. Cancer Discov. 11 (7), 1808–1825. 10.1158/2159-8290.Cd-20-0119 33653692 PMC8292164

[B186] Zhang Y. Zuo T. McVicar A. Yang H. L. Li Y. P. Chen W. (2022a). Runx1 is a key regulator of articular cartilage homeostasis by orchestrating YAP, TGFβ, and Wnt signaling in articular cartilage formation and osteoarthritis. Bone Res. 10 (1), 63. 10.1038/s41413-022-00231-y 36307389 PMC9616925

[B187] Zhao B. Lei Q.-Y. Guan K.-L. (2008). The Hippo–YAP pathway: new connections between regulation of organ size and cancer. Curr. Opin. Cell Biol. 20 (6), 638–646. 10.1016/j.ceb.2008.10.001 18955139 PMC3296452

[B188] Zhao F. Zhang L. Wei M. Duan W. Wu S. Kasim V. (2022b). Mechanosensitive ion channel Piezo1 signaling in the Hall-marks of cancer: structure and functions. Cancers (Basel) 14 (19), 4955. 10.3390/cancers14194955 36230880 PMC9563973

[B189] Zhao Q. Zhou H. Li X. Xiao B. (2019). The mechanosensitive Piezo1 channel: a three-bladed propeller-like structure and a lever-like mechanogating mechanism. Febs J. 286 (13), 2461–2470. 10.1111/febs.14711 30500111

[B190] Zhao W. Wei Z. Xin G. Li Y. Yuan J. Ming Y. (2021). Piezo1 initiates platelet hyperreactivity and accelerates thrombosis in hypertension. J. Thromb. Haemost. 19 (12), 3113–3125. 10.1111/jth.15504 34411418

[B191] Zhao X. Kong Y. Liang B. Xu J. Lin Y. Zhou N. (2022a). Mechanosensitive Piezo1 channels mediate renal fibrosis. JCI Insight 7 (7), e152330. 10.1172/jci.insight.152330 35230979 PMC9057604

[B192] Zhao Y. Y. Guo M. M. Zhao F. Q. Liu Q. Wang X. (2023). Colonic stem cells from normal tissues adjacent to tumor drive inflammation and fibrosis in colorectal cancer. Cell Commun. Signal. 21 (1), 186. 10.1186/s12964-023-01140-1 37528407 PMC10391886

[B193] Zhong M. Komarova Y. Rehman J. Malik A. B. (2018). Mechanosensing Piezo channels in tissue homeostasis including their role in lungs. Pulm. Circ. 8 (2), 2045894018767393. 10.1177/2045894018767393 29521167 PMC6024292

[B194] Zhou B. P. Liao Y. Xia W. Spohn B. Lee M. H. Hung M. C. (2001). Cytoplasmic localization of p21Cip1/WAF1 by Akt-induced phosphorylation in HER-2/neu-overexpressing cells. Nat. Cell Biol. 3 (3), 245–252. 10.1038/35060032 11231573

[B195] Zhou W. Liu X. van Wijnbergen J. W. M. Yuan L. Liu Y. Zhang C. (2020). Identification of PIEZO1 as a potential prognostic marker in gliomas. Sci. Rep. 10 (1), 16121. 10.1038/s41598-020-72886-8 32999349 PMC7528027

[B196] Zhu D. Zhang G. Guo X. Wang Y. Liu M. Kang X. (2021). A new hope in spinal degenerative diseases: piezo1. Biomed. Res. Int. 2021, 6645193. 10.1155/2021/6645193 33575334 PMC7857891

[B197] Zhu T. Guo J. Wu Y. Lei T. Zhu J. Chen H. (2023). The mechanosensitive ion channel Piezo1 modulates the migration and immune response of microglia. iScience 26 (2), 105993. 10.1016/j.isci.2023.105993 36798430 PMC9926228

[B198] Zhu Z. Li W. Gong M. Wang L. Yue Y. Qian W. (2022). Piezo1 act as a potential oncogene in pancreatic cancer progression. Life Sci. 310, 121035. 10.1016/j.lfs.2022.121035 36208662

